# Bipyraloxifene – a modified raloxifene vector against triple-negative breast cancer†

**DOI:** 10.1039/d4md00051j

**Published:** 2024-04-03

**Authors:** Aleksandr Kazimir, Tom Götze, Blagoje Murganić, Sanja Mijatović, Danijela Maksimović-Ivanić, Evamarie Hey-Hawkins

**Affiliations:** a Institute of Inorganic Chemistry, Faculty of Chemistry and Mineralogy, Leipzig University Johannisallee 29 04103 Leipzig Germany hey@uni-leipzig.de; b Institute of Nuclear Sciences “Vinča”, University of Belgrade 12-14 Mike Petrovića Street Belgrade 11351 Serbia; c Department of Immunology, Institute for Biological Research “Siniša Stanković”, National Institute of Republic of Serbia, Belgrade University Bul. despota Stefana 142 Belgrade 11060 Serbia nelamax@ibiss.bg.ac.rs

## Abstract

Raloxifene, a selective oestrogen receptor modulator (SERM), has demonstrated efficacy in the prevention and therapy of oestrogen receptor-positive (ER+) breast cancer, with some degree of effectiveness against triple-negative forms. This suggests the presence of oestrogen receptor-independent pathways in raloxifene-mediated anticancer activity. To enhance the potential of raloxifene against the most aggressive breast cancer cells, hybrid molecules combining the drug with a metal chelator moiety have been developed. In this study, we synthetically modified the structure of raloxifene by incorporating a 2,2′-bipyridine (2,2′-bipy) moiety, resulting in [6-methoxy-2-(4-hydroxyphenyl)benzo[*b*]thiophen-3-yl]-[4-(2,2′-bipyridin-4′-yl-methoxy)phenyl]methanone (bipyraloxifene). We investigated the cytotoxic activity of both raloxifene and bipyraloxifene against ER+ breast adenocarcinomas, glioblastomas, and a triple-negative breast cancer (TNBC) cell line, elucidating their mode of action against TNBC. Bipyraloxifene maintained a mechanism based on caspase-mediated apoptosis but exhibited significantly higher activity and selectivity compared to the original drug, particularly evident in triple-negative stem-like MDA-MB-231 cells.

## Abstract 2

The synthetic combination of raloxifene, a selective estrogen receptor modulator, with a 2,2′-bipyridine moiety results in bipyraloxifene, a potential drug with enhanced activity against triple negative breast cancer.

## Graphical abstract

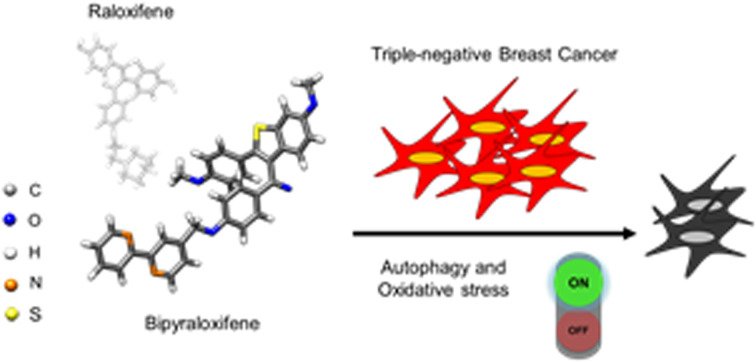

## Introduction

Selective oestrogen receptor modulators (SERMs) are a class of compounds that exhibit both agonistic and antagonistic effects on the oestrogen receptor (ER)^1^ through non-covalent binding to its ligand-binding domain (LBD).^2^ These versatile compounds have found extensive applications in the treatment of various oestrogen-related diseases (*e.g.* osteoporosis and breast cancer), showcasing their ability to tailor their mode of action depending on the target tissue.^3^ Specifically SERMs are known for their application in prevention and treatment of hormone-receptor positive (HR+) breast cancer, where luminal A is the most frequent subtype.^4^ However, the efficacy of such treatment diminishes in the case of triple-negative breast cancer (TNBC), an aggressive and heterogeneous subtype characterised by the absence of expression of oestrogen receptor α (ERα), progesterone receptor (PR), and human epidermal growth factor receptor 2 (HER2).^5,6^ Interestingly, a certain subgroup of TNBC expresses oestrogen receptor β (ERβ), whose controversial role makes it a potential target for cancer therapy.^7^

**Fig. 1:**
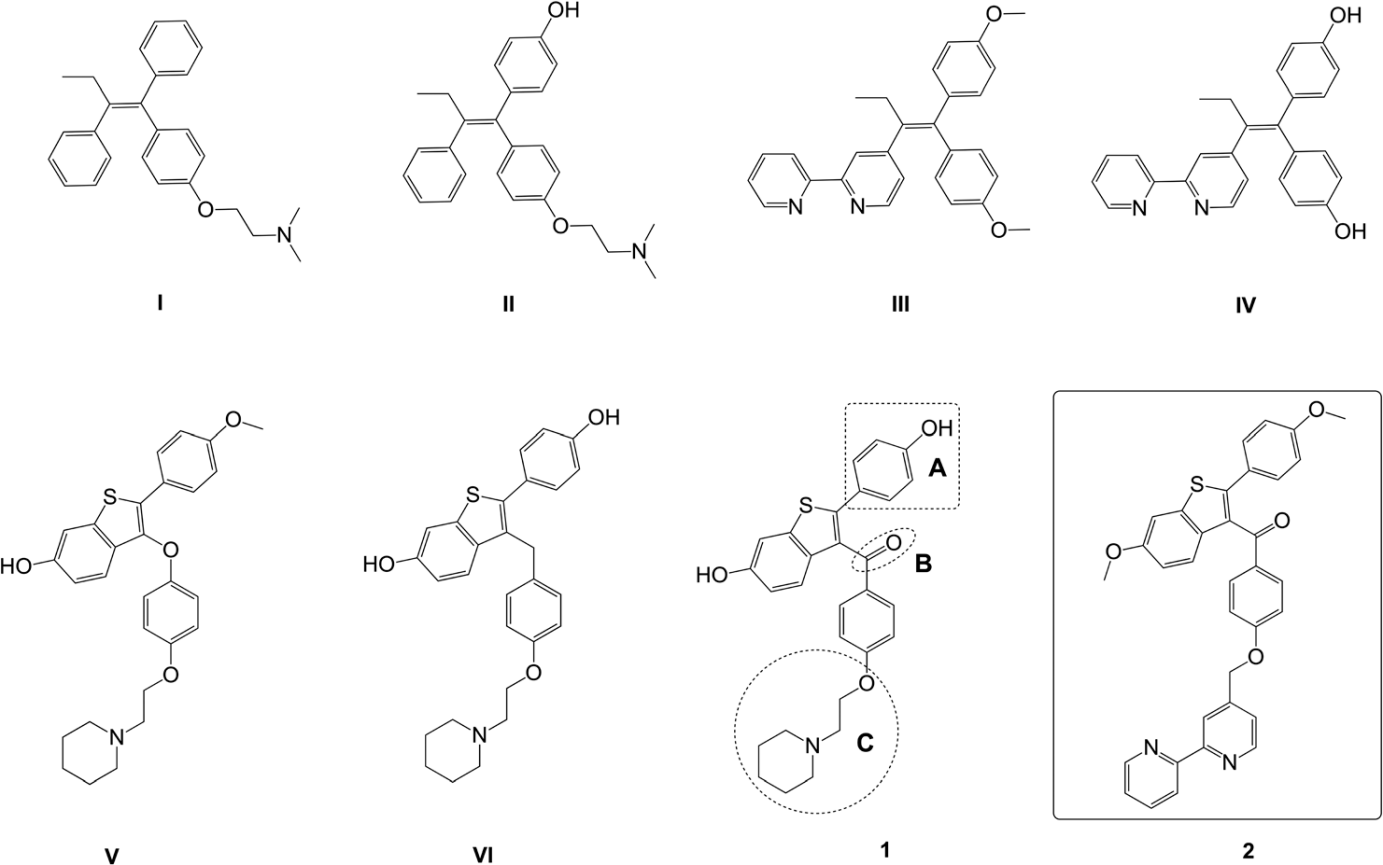
Combination of selective oestrogen receptor modulators (SERMs) with a 2,2′-bipyridine moiety. First generation SERMs: tamoxifen (I),^12^ 4-hydroxytamoxifen (II),^18^ 4-[1,1-bis(4-methoxyphenyl)but-1-en-2-yl]-2,2′-bipyridine (III),^19,20^ and 4-[1,1-bis(4-hydroxyphenyl)but-1-en-2-yl]-2,2′-bipyridine (IV).^19^ Raloxifene-inspired second generation SERMs: arzoxifene (V),^21^ 2-(4-hydroxyphenyl)-3-{4-[2-(piperidin-1-yl)ethoxy]benzyl}benzo[*b*]thiophen-6-ol (VI),^22^ raloxifene (1),^23^ [6-methoxy-2-(4-hydroxyphenyl)benzo[*b*]thiophen-3-yl]-[4-(2,2′-bipyridin-4′-yl-methoxy)phenyl]methanone (bipyraloxifene) (2). Compounds 1 and 2, raloxifene and bipyraloxifene, respectively, are explored in this study.

Delving deeper into understanding of the role of the endocrine system in the development of TNBC tumours created the possibility to re-evaluate SERMs for treatment of this subtype of breast cancer.^8,9^ For instance, it was shown that a specific isoform of ERα (*e.g.* ERα36) mediates the oestrogen signalling pathway participating in specific transcriptomic signatures of TNBC.^10,11^ 4-Hydroxytamoxifen (II, Fig. 1) is an active metabolite of tamoxifen (I, Fig. 1)^12,13^ (SERM of the first generation) serving as an antagonist of ERα in breast tissue. However, it has a contentious impact in TNBC environment where it serves as an agonist for G-protein coupled ER (GPER) showing its carcinogenic role.^14,15^ The SERM raloxifene has demonstrated the ability to reduce TNBC tumour growth *in vivo* and provoke tumour regression *via* decreasing the expression of epidermal growth factor receptor (EGFR).^16^ Additionally, O'Donnell *et al.* discovered raloxifene's potential to affect viability of TNBC through interaction with the aryl hydrocarbon receptor (AhR).^17^ However, the application of raloxifene in TNBC therapy requires an enhancement in order to boost its efficacy.

The raloxifene structure was modified leading to more potent analogues with diverse mechanism of action (Fig. 1). For instance arzoxifene (V) bearing a methoxy group (1, position A) and an oxygen bridge (1, position B) showed an even better efficiency than raloxifene in the prevention of mammary breast cancer induced in rats.^21^ Interestingly, an alkylene (CH_2_) linker (Fig. 1, 1) at position B increases the flexibility of the chain and improves the inhibitory activity of raloxifene analogue VI against gut microbial β-glucuronidase (GUS) enzymes, which are proposed to play a role in the pathogenesis of breast cancer.^22^ A more prominent study in the context of TNBC has demonstrated that a combination of raloxifene and the tyrosine kinase inhibitor gefitinib increases the cytotoxicity towards TNBC cell cultures by targeting different signalling pathways.^24,25^ However, the use of a combination of different drugs requires careful consideration. In the therapy of breast cancer there are some known effective combinations of drugs;^26^ however, due to the heterogeneous nature of breast cancer and its dependence on numerous factors, it is difficult to establish a universal protocol to use a certain combination of drugs.^27^ Additionally, different pharmacokinetic and synergetic properties make the search of drug combinations complicated,^28^ while a multiple therapeutic effect could be achieved within a single molecule of a hybrid drug.^29–31^

To aid the improvement of an anticancer mechanism of raloxifene we have incorporated the strong chelating unit 2,2′-bipyridine (2,2′-bipy). The potential application of a 2,2′-bipy-containing compound in chelation therapy arises from the ability to coordinate metals that are essential for the metabolism (*e.g.* Fe, Cu, Zn), which are typically present in excess in cancer cells due to the accelerated metabolism of cancerous compared to the regular tissue.^32–36^ Especially interesting is the application of the 2,2′-bipy moiety as an iron chelator, due to the fact that cancer cells require a higher amount of iron for rapid DNA synthesis and tumour growth.^37^ Recently it was reported that mesenchymal like TNBC cells such as MDA-MB-231 display significantly more sensitivity to iron deprivation than less advanced forms of transformed cells and thus represent a good target for treatment with iron chelators.^38^ Moreover, due to the excess iron, the tumour demonstrates increased production of reactive oxygen species (ROS) inducing damage of DNA and further cancer development.^39^ Essentially the so-called phosphoinositide 3-kinases/protein kinase B (PI3K/PKB) pathway responsible for growth and metabolism of metastatic phenotype of TNBC can be effectively inhibited by iron chelators.^40^ The 2,2′-bipy moiety is known as an intracellular iron chelator.^41^ Furthermore, recent studies have shown that mono-, bis-, and trisbipyridine molecules exhibit cytotoxicity against leukaemia and lymphoma, with cytotoxicity improving upon the addition of 2,2′-bipyridine moieties.^42^ Additionally, the 2,2′-bipy unit is able to induce DNA cleavage *via* intercalation.^43^ Therefore, inspired by these interesting results we took 2,2′-bipy as a promising moiety to incorporate a dual therapeutic effect into the raloxifene molecule.

Previously we have combined a tamoxifen-inspired structure with a 2,2′-bipy unit (compounds III and IV, Fig. 1) and demonstrated that incorporation of a 2,2′-bipy moiety increases not only the cytotoxic activity towards HR+ breast cancer cell lines, such as U251, MCF-7 and MDA-MB-361, but also against TNBC (MDA-MB-231). Moreover, 2,2′-bipy-modified tamoxifen derivatives III and IV activate autophagy and antioxidant effects.^19,20,44^ Apparently, the 2,2′-bipy unit improves the cytotoxic potential of SERMs enabling a hormone-independent mechanism of action.

Therefore, in this study taking together the advantages of raloxifene and the potential of chelation therapy we explored the application of a raloxifene-inspired structure combined with 2,2′-bipy towards TNBC *in vitro*.

## Results and discussion

### Synthesis and characterisation

A suitable way for the synthetic modification of the raloxifene-based structure was firstly published by Schmid *et al.*^45^ This approach involves nucleophilic aromatic substitution of fluoride in compound f using the different oxygen-, sulfur- or nitrogen-based nucleophiles. The oxygen and sulfur nucleophiles were deprotonated by NaH, while for N-based nucleophiles KF/Al_2_O_3_ was used.^45^ A modified synthesis was employed for bipyraloxifene (2).

**Scheme 1:**
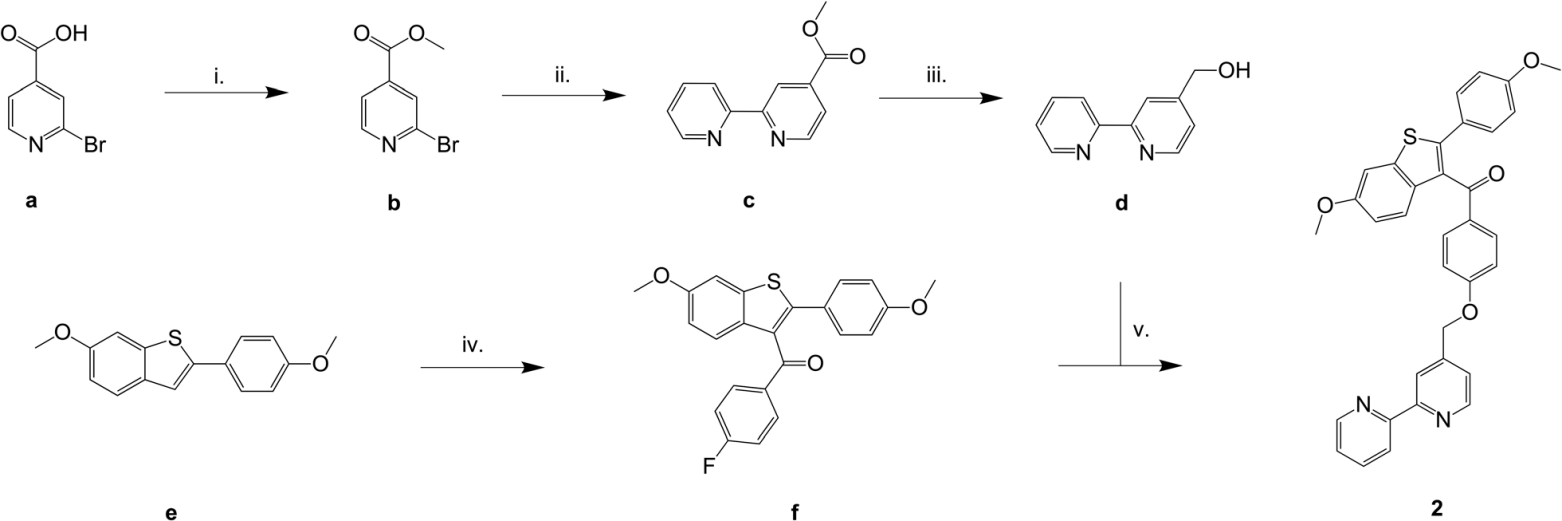
Synthesis of bipyraloxifene (2). i. 1-Ethyl-3-(3-dimethylaminopropyl)carbodiimide hydrochloride (EDC·HCl), 0 °C → rt, DCM/MeOH; ii. 2-(tributylstannyl)pyridine, [Pd(PPh_3_)_4_], toluene, reflux; iii. LiBH_4_, THF, 0 °C → rt; iv. 4-fluorobenzoylchloride, AlCl_3_/DCM, 0 °C → rt, 12 h, HCl; v. NaH, THF, 0 °C → rt.

Bipyraloxifene (2) was prepared in five steps (Scheme 1). The 2,2′-bipy-containing methanol derivative (d) was prepared from commercially available 2-bromoisonicotinic acid (a), which was first converted to the methyl ester (b), then coupled with 2-(tributylstannyl)pyridine (Stille coupling) to obtain 4-carboxymethyl-2,2′-bipyridine (c). The ester group of c was reduced with LiBH_4_ to generate 4-(2-hydroxymethyl)-2,2′-bipyridine (d, Scheme 1). In parallel, the raloxifene moiety ([4-fluorophenyl-5-methoxy-2-(4-methoxyphenyl)-benzo[*b*]thiophen-3-yl]methanone, f, Scheme 1) was prepared from 6-methoxy-2-(4-methoxyphenyl)benzo[*b*]thiophene (e) as starting material *via* a Friedel–Crafts acylation in DCM. In the last step, 2,2′-bipy alcohol (d) was deprotonated with NaH in THF and reacted with f in a nucleophilic aromatic substitution resulting in [6-methoxy-2-(4-hydroxyphenyl)-benzo[*b*]thiophen-3-yl]-[4-(2,2′-bipyridin-4′-yl-methoxy)phenyl]methanone (bipyraloxifene) (2).

Bipyraloxifene was fully characterised by ^1^H and ^13^C{^1^H} NMR, UV-vis and IR spectroscopy and mass spectrometry (details are given in the ESI†). For *in vitro* tests, stock solutions of raloxifene (1) and bipyraloxifene (2) in DMSO were prepared and stored at +4 °C. To assure the stability of bipyraloxifene (2), ^1^H NMR spectra were recorded in water-containing DMSO-d_6_ in air, confirming that the compound is stable for at least two months.

### Cytotoxicity study

The impact of raloxifene (1) and bipyraloxifene (2) towards breast cancer cell lines exhibiting diverse hormone and HER2 expression profiles was evaluated. To gain deeper insights into the significance of hormone receptor expression in the context of breast cancer cells, the screening encompassed well-known cell lines such as MCF-7, MDA-MB-231, and MDA-MB-361. Additionally, we broadened the scope by including a non-breast cancer cell line, U251 human glioblastoma, which expresses ER. The drug selectivity towards the malignant phenotype was further assessed by the ratio between the sensitivity of specific cancer cell lines to the applied doses and the response of normal peritoneal exudate cells (PEC). Cell viability was determined by measuring the total mitochondrial respiration and number of adherent cells in cultures, using 3-(4,5-dimethylthiazol-2-yl)-2,5-diphenyltetrazolium bromide (MTT) and crystal violet (CV) assays, respectively (Table 1).

**Table 1:** IC_50_ values (μM) of raloxifene (1) and bipyraloxifene (2) from MTT and CV assays after 72 h incubation shown as mean together with one standard deviation (mean ± SD)

Compounds	Assays	U251	MCF-7	MDA-MB-361	MDA-MB-231	PEC
		μM	μM	μM	μM	μM
1	MTT	19.25 ± 1.06	16.80 ± 0.99	22.15 ± 1.48	19.1 ± 1.70	18.3 ± 0.28
	CV	23.35 ± 1.34	21.45 ± 2.76	38.9 ± 0.85	22 ± 0.85	20.35 ± 0.07
2	MTT	2.2 ± 0.35	2.15 ± 0.35	6.3 ± 0.001	1.6 ± 0.14	15.4 ± 0.14
	CV	2.6 ± 0.28	2.4 ± 0.28	>100	1.5 ± 0.28	18.95 ± 0.07

The cytotoxic potential of bipyraloxifene (2) demonstrates a significant multiplicative improvement towards the tested cancer lines when compared to the parental drug raloxifene (1). Interestingly, the applied chemical modification resulted in an increased selectivity towards the TNBC cancer cell line MDA-MB-231, as demonstrated by the selectivity index (SI) of approximately 12.6 determined by the CV test, or 9.5 by MTT.

The improvement in cytotoxicity of bipyraloxifene may be closely related to its iron chelating properties. In concordance with what was mentioned above, stem like TNBC cells, MDA-MB-231, showed elevated susceptibility to iron deprivation being a good candidate for the treatment with iron chelators.^38^

In keeping with this, treatment with bipyraloxifene (2) may exhibit dual activity against the aforementioned cell line, partially through metal chelating properties developed *via* applied structural intervention, as well as in relation to its raloxifene-like effect on intracellular signaling pathways responsible for tumour proliferation.

Compound 2 is developed from an original drug designed to inhibit ER. On the other hand, raloxifene has been described in the literature to affect even TNBC cells in an ER-independent manner through interaction with the AhR, leading to increased apoptosis of these cells.^38^ Apart from this, a decrease in epidermal growth factor receptor (EGFR) expression was observed in response to raloxifene. While this receptor mediated proliferative signaling, its suppression resulted in abolished malignant potential of the cells. Additionally to justify the targeting mechanism of raloxifene and bipyraloxifene we have carried out the docking of raloxifene and bipyraloxifene in several receptors which might be potential targets in TNBC therapy. As raloxifene is known to exhibit high affinity to ERα and ERβ, we firstly estimated and compared the binding energies of the synthesised drug towards these receptors. We found out an only insignificant decrease of the binding affinities of 2 towards ERα and ERβ compared to raloxifene (ERα: −12.5 kcal mol^−1^ for 1 and −11.0 kcal mol^−1^ for 2; ERβ: −12.6 kcal mol^−1^ for 1 and −11.9 kcal mol^−1^ for 2). This indicates that bipyraloxifene can exhibit potential binding to both receptors and act as a SERM. Interestingly, compound 2 showed even higher affinity to ERβ than to ERα, as the first one can be a promising target in TNBC treatment.^46^ ERβ participates in the interaction with the EGFR, where it was assumed that binding of raloxifene to ERβ decreases the signaling of EGFR suppressing tumour progress.^47^ We have, therefore, additionally considered *in silico* binding affinities of bipyraloxifene to EGFR and found that both drugs 1 and 2 have similar binding energies indicating EGFR as a potential target. Furthermore raloxifene serves as a binding ligand to AhR inducing apoptosis in TNBC.^48^ Docking of compound 2 into this receptor demonstrated that incorporation of the 2,2′-bipy unit decreases the binding abilities of this molecule to this receptor (−7.7 kcal mol^−1^ for 1 and −4.6 kcal mol^−1^ for 2) indicating a rather low probability to interact with AhR (see ESI,† Docking).

### Flow cytometry

**Fig. 2:**
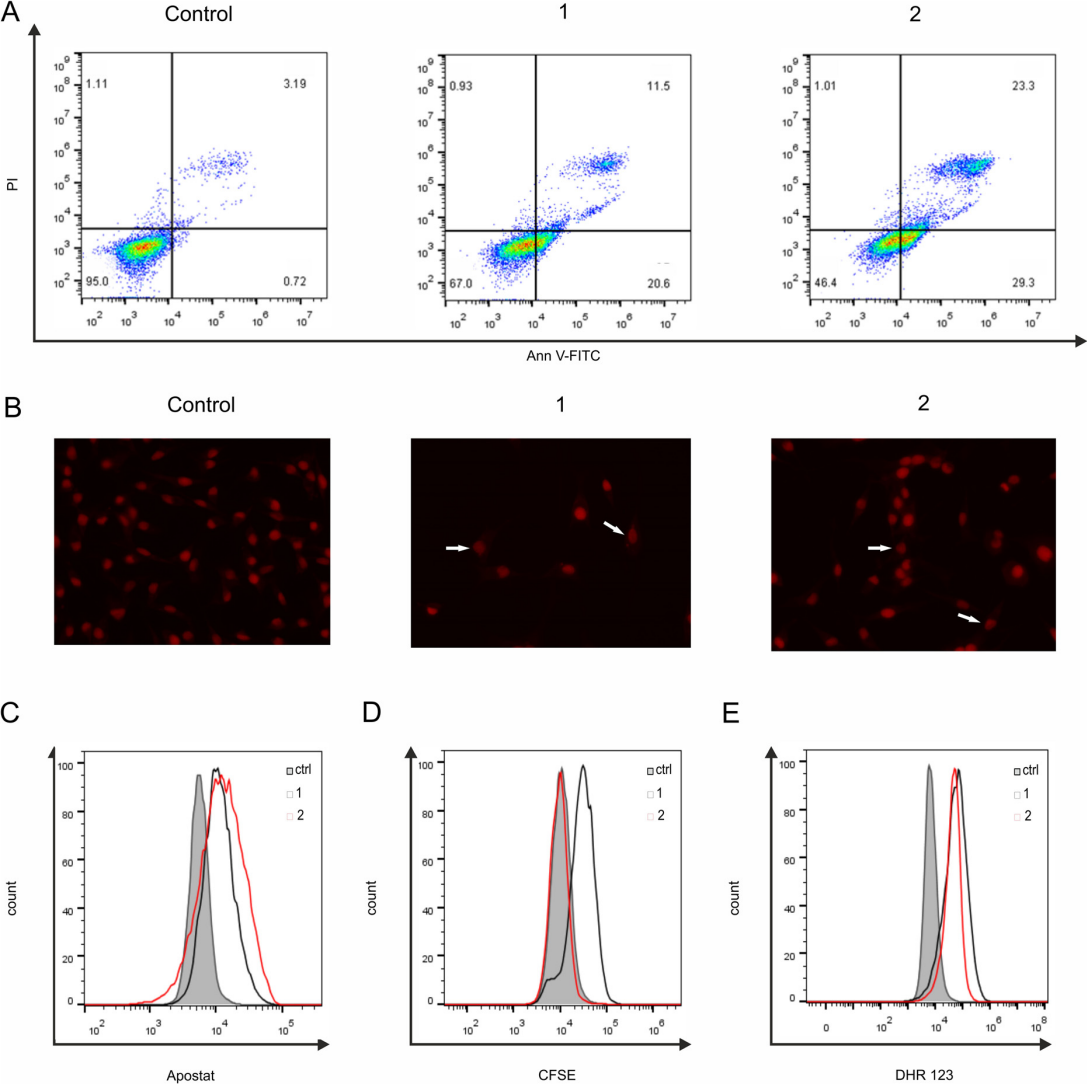
Bipyraloxifene (2) induced caspase-dependent apoptosis of MDA-MB-231 cells. Cells were exposed to an IC_50_ dose of 1 or 2 for 72 h and analysed by flow cytometry: (A) apoptosis detection (Annexin V/PI staining), (B) morphology of nuclei: arrows indicate apoptotic cells with irregular cell nuclei (PI staining, 400× magnification), (C) caspase activation (Apostat staining), (D) cell proliferation (carboxyfluorescein diacetate succinimidyl ester staining (CFSE)), (E) reactive oxygen and nitrogen species (ROS/RNS) production (dihydrorhodamine 123 (DHR 123) staining). Representative histograms from three independent experiments are shown.

The mode of the experimental drug action *vs.* the original compound was explored in MDA-MB-231 as the mostly affected triple-negative cell line. Examination of cell death through Annexin V/propidium iodide (Ann/PI) double staining revealed a substantial induction of apoptosis following a 72-hour exposure of MDA-MB-231 cells to an IC_50_ dose of either raloxifene (1) or bipyraloxifene (2) (Fig. 2A). Notably, the accumulation of early and late apoptotic cells was more pronounced in cultures treated with bipyraloxifene (2). Fluorescent microscopy of cells exposed to raloxifene (1) or bipyraloxifene (2) for the indicated incubation period, followed by fixation and staining with PI, confirmed the prevalence of apoptosis at the morphological level, detecting numerous nuclei exhibiting condensed chromatin, abnormal size, and shape (Fig. 2B). Both compounds initiated the apoptotic process in a caspase-dependent manner (Fig. 2C), as previously observed in cancer cell lines derived from various tumour types.^23,45,49^ Importantly, the significant induction of apoptosis was not correlated with suppressed cell proliferation, as assessed by CFSE (Fig. 2D).

Furthermore, an intense cytocidal effect of raloxifene (1) and bipyraloxifene (2), a notable oxidative burst, measured by dihydrorhodamine 123 (DHR) staining, was observed, suggesting the involvement of reactive oxygen and nitrogen species in the process of a cell structure damage and death induction (Fig. 2E). This result contrasts with previously published data demonstrating the antioxidative features of raloxifene (1), indicating the dual nature of raloxifene depending on cell specificity.^50,51^

**Fig. 3:**
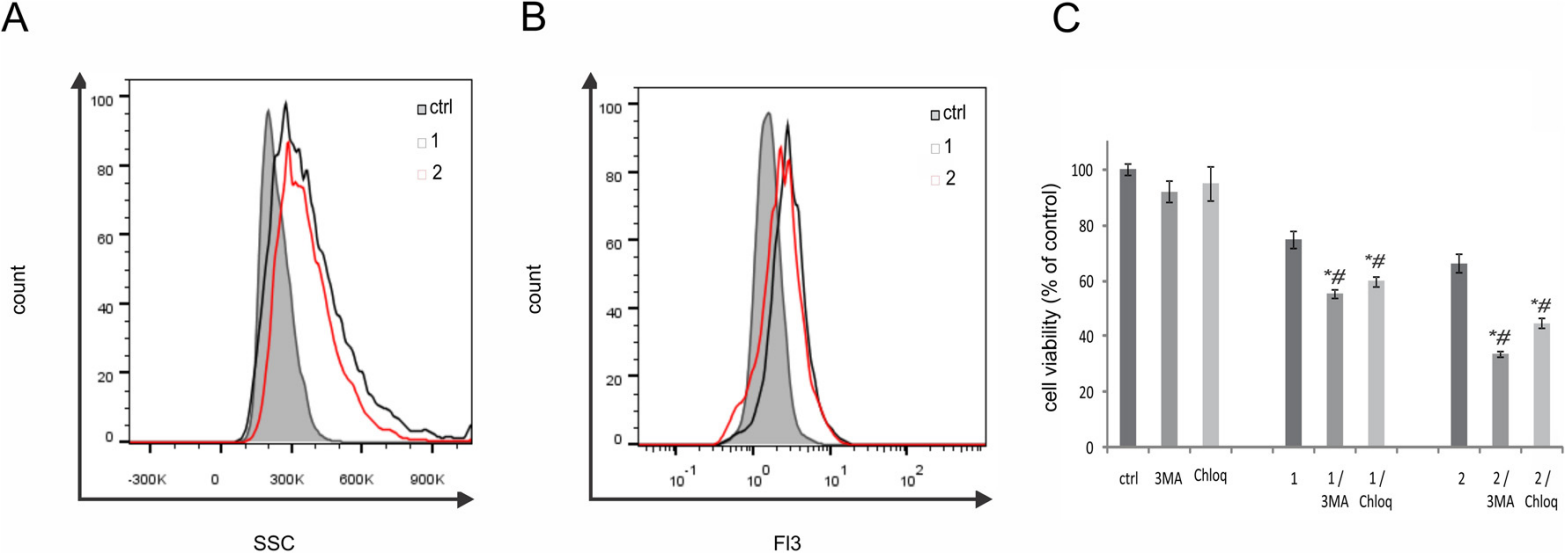
Bipyraloxifene (2) induced cytoprotective autophagy. Cells were exposed to an IC_50_ dose of raloxifene (1) or bipyraloxifene (2) for 72 h and analysed by flow cytometry: (A) cell granularity, (B) autophagy detection (acridine orange staining), (C) cell viability determination in concomitant treatment with 3-MA or chloroquine by CV assay. *p* < 0.05. *p* < 0.05 in comparison to untreated control (*) or cells treated with compounds 1 or 2 (^#^).

Concurrently with the observed increase in the granularity of the cellular cytoplasm through flow cytometry, supravital staining of the same cell line with acridine orange after a 72-hour treatment with raloxifene (1) or bipyraloxifene (2) resulted in a significant augmentation in the presence of autophagosomes, indicating a heightened autophagic process in response to the treatment (Fig. 3A). Inhibition of the autophagic process by specific inhibitors, 3-methyladenine (3-MA) or chloroquine, markedly intensified the effects of both compounds 1 and 2, emphasising that autophagy opposes apoptosis and does not function as programmed cell death type 2 in response to raloxifene (1) or bipyraloxifene (2) (Fig. 3B and C).

The potential of raloxifene (1) to trigger the autophagic process is well-established. Considering that autophagy can serve as both a protective and destructive process depending on the rate of intracellular damage, it is not surprising that in this study its presence is associated with the cell's attempt to overcome apoptotic signals.^52^

Overall, bipyraloxifene (2) demonstrates an almost identical mechanism of action as the original drug, but with a significantly enhanced cytotoxic potential, that can be fully explained by its chelating properties as well as raloxifene like off-targets involvement.

## Conclusions

Raloxifene, a selective oestrogen receptor modulator, showcases a versatile mode of action, highlighting its potential as an anticancer agent against both hormone-dependent and triple-negative breast cancer cell lines. The incorporation of the 2,2′-bipy unit in bipyraloxifene (2) enhances the cytotoxic activity across all tested cell lines, with a particularly heightened sensitivity observed in the most advanced triple-negative breast cancer cells (MDA-MB-231). It can be speculated that this improvement might be attributed to both, iron depletion and interference with ERα independent intracellular targets. Notably, bipyraloxifene (2) preserves the mechanism of original drug action (1) in terms of induction of caspase-dependent apoptosis, oxidative stress, and the initiation of cytoprotective autophagy. These findings underscore the potential of bipyraloxifene (2) as a promising candidate for the design of hybrid molecules in anticancer drug development. Finally, the incorporation of the 2,2′-bipy unit into the raloxifene structure enables the combination of bipyraloxifene with certain other metal-based complexes that have shown promising anticancer properties (*e.g.*, PtCl_2_), making it a good platform for future studies.

## Experimental section

### Reagents and cells

Reagents and cells were sourced from the following manufacturers: dimethyl sulfoxide (DMSO), crystal violet (CV), 3-methyladenine (3-MA), phosphate-buffered saline (PBS), propidium iodide (PI), carboxyfluorescein diacetate succinimidyl ester (CFSE), fluorescent mounting medium, and acridine orange (AO) were obtained from Sigma (St. Louis, MO, USA). Paraformaldehyde (PFA) was acquired from SERVA Electrophoresis GmbH (Heidelberg, Germany). 3-(4,5-Dimethylthiazol-2-yl)-2,5-diphenyltetrazolium bromide (MTT) was obtained from AppliChem (Darmstadt, Germany). Culture medium RPMI-1640 and fetal bovine serum (FBS) were obtained from Capricorn Scientific GmbH (Ebsdorfergrund, Germany). Penicillin–streptomycin solution was purchased from Biological Industries (Cromwell, CT, USA). Annexin V-FITC (AnnV) was acquired from BD (Pharmingen, San Diego, CA, USA). ApoStat was obtained from R&D Systems (Minneapolis, MN, USA). HEPES (4-(2-hydroxyethyl)-1-piperazineethanesulfonic acid)-buffered RPMI (Roswell Park Memorial Institute)-1640 medium, chloroquine, and dihydrorhodamine 123 (DHR 123) were purchased from Thermo Fisher Scientific (Waltham, MA, USA). Cell lines (human malignant glioma U251; human breast adenocarcinoma MCF-7, MDA-MB-361, and MDA-MB-231) were obtained from the American Type Culture Collection (ATCC, Manassas, VA, USA). Mouse peritoneal exudate cells were isolated and treated exactly as described in Kazimir *et al.*^20^

Cell lines were cultured in HEPES-buffered RPMI-1640 medium supplemented with 10% heat-inactivated fetal calf serum (FCS), 2 mM l-glutamine, 0.01% sodium pyruvate, and antibiotics (penicillin 100 units per mL, streptomycin 100 μg mL^−1^). The cells were maintained at 37 °C in a humidified atmosphere with 5% CO_2_. For viability determination, MCF-7, MDA-MB-231, MDA-MB-361, and U251 were seeded at a density of 7 × 10^3^, 4 × 10^3^, 6 × 10^3^, and 2 × 10^3^, respectively, in 96-well plates. For flow cytometric analyses, MDA-MB-231 cells were seeded at a density of 1 × 10^5^ cells per well in 6-well plates. For morphological nuclei assessment MDA-MB-231 cells were seeded at a density of 1 × 10^4^ cells per well in 8-well chamber slides.

The compounds were initially dissolved in DMSO at a concentration of 20 mM and stored at −20 °C as stock solutions. Prior to use, working solutions were prepared by diluting the DMSO stock with cell medium.

### Determination of cell viability (MTT and CV assays)

Cell lines were seeded overnight and treated with a range of doses of raloxifene (1) or bipyraloxifene (2) for 72 h. After incubation, the cells were washed, fixed, and subjected to MTT and CV assays to determine cell viability as described previously.^53^ Alternatively, cells were treated with an IC_50_ dose of raloxifene (1) or bipyraloxifene (2) in parallel with autophagy inhibitors 3-MA (1 mM) or chloroquine (10 μM) for 72 h; viability was assessed by MTT test.

### Annexin V (AnnV)/propidium iodide (PI), ApoStat, and acridine orange (AO) staining

Cells were treated with an IC_50_ dose of raloxifene (1) or bipyraloxifene (2) for 72 h; apoptosis was detected using Annexin V/propidium iodide staining (15 μg mL^−1^ Annexin V (AnnV), propidium iodide (PI) staining). Caspase activity was assessed by incubating cells with pan-caspase inhibitor ApoStat for 30 min at 37 °C. The presence of autophagic vesicles as a marker of autophagy was evaluated using 1 μg mL^−1^ acridine orange (AO) stain for 15 min at 37 °C. Complete procedures were described in Braun *et al.*^53^ Cells were analysed using flow cytometry (CytoFLEX Flow Cytometer, Beckman Coulter, Life Sciences, Indianapolis, IN, USA or CyFlow® Space Partec using the PartecFloMax® software (Münster, Germany)).

### Carboxyfluorescein diacetate succinimidyl ester (CFSE) staining

Cell proliferation was analysed using CFSE staining. Cells were stained with 1 μM CFSE for 10 min at 37 °C, seeded, and treated with an IC_50_ dose of raloxifene (1) or bipyraloxifene (2) for 72 h. At the end of incubation (72 h), cells were trypsinised, washed, and re-suspended in PBS. The analysis was done using flow cytometry (CytoFLEX Flow Cytometer, Beckman Coulter, Life Sciences, Indianapolis, IN, USA).

### Measurement of ROS/RNS generation

Production of reactive oxygen and nitrogen species (ROS/RNS) was detected by pre-staining cells with 1 μM DHR for 20 min at 37 °C, after treatment with raloxifene (1) or bipyraloxifene (2) for 72 h. Cells were then washed with PBS, trypsinised, and analysed using flow cytometry (CytoFLEX Flow Cytometer, Beckman Coulter, Life Sciences, Indianapolis, IN, USA).

### PI staining on chamber slides

MDA-MB-231 cells were treated with the IC_50_ concentration of raloxifene (1) or bipyraloxifene (2) for 72 h. After the incubation period, cells were fixed with 4% paraformaldehyde (PFA) for 15 min at room temperature and stained with a solution of propidium iodide (PI) (50 μg mL^−1^) containing 0.1% Triton X-100, 0.1 mM EDTA (pH 8.0), and RNase (85 μg mL^−1^) in phosphate-buffered saline (PBS) for 2 min. Subsequently, the slides were washed in PBS and mounted using a mounting medium to prepare the cells for fluorescence microscopy. The slides were examined using a Zeiss AxioObserver Z1 inverted fluorescence microscope (Carl Zeiss AG, Oberkochen, Germany).

### Statistical analysis

The data presented represent the mean ± SD of at least three independent experiments. Student's *t*-test was used to evaluate the significance between groups, and *p*-values less than 0.05 were considered statistically significant.

### Synthesis

Materials, methods and procedure are given in the ESI.† The synthesis of bipyraloxifene (2) including the preparation of the starting materials involves five steps which are reported in the ESI.†

## Author contributions

Conceptualisation, E. H.-H., A. K. and D. M.-I.; methodology, A. K., T. G., B. M., D. M.-I. and S. M.; validation, A. K., T. G., B. M., D. M.-I. and S. M.; formal analysis, A. K., T. G., B. M., D. M.-I. and S. M.; investigation, A. K., T. G., B. M., S. M. and D. M.-I.; resources, E. H.-H., D. M.-I. and S. M.; data curation, A. K., E. H.-H., D. M.-I. and S. M.; writing – original draft preparation, A. K.; writing – review and editing, A. K., T. G., E. H.-H., D. M.-I., B. M. and S. M.; visualisation, A. K., T. G., B. M., D. M.-I. and S. M.; supervision, E. H.-H., D. M.-I. and S. M.; project administration, E. H.-H., D. M.-I. and S. M.; funding acquisition, E. H.-H., D. M.-I. and S. M. All authors have read and agreed to the published version of the manuscript descriptions.

## Conflicts of interest

There is no conflict to declare.

## Supplementary Material

MD-015-D4MD00051J-s001
