# Supplementary material for: Bipyraloxifene – a modified raloxifene vector against triple-negative breast cancer
Source: RSC Med Chem. 2024 Apr 3;15(6):1921–8. doi: 10.1039/d4md00051j (PMC11187558; doi:10.1039/d4md00051j)
Supplement: MD-015-D4MD00051J-s001 [file MD-015-D4MD00051J-s001.pdf]

Electronic Supplementary Material (ESI) for RSC Medicinal Chemistry.
This journal is © The Royal Society of Chemistry 2024

Supporting Information

Bipyraloxifene - A Modified Raloxifene Vector against Triple-Negative Breast Cancer

Aleksandr Kazimir[a], Tom Götze[a], Blagoje Murganić[b], Sanja Mijatović[c], Danijela Maksimović-Ivanić[c]* and Evamarie Hey-Hawkins[a]*

[a]Institute of Inorganic Chemistry, Faculty of Chemistry and Mineralogy, Leipzig University, Leipzig, Germany; A. Kazimir, T. Götze New address: Institute for Drug Discovery, Leipzig University, Brüderstraße 34, 04103 Leipzig, Germany

[b]Institute of Nuclear Sciences "Vinča", University of Belgrade, 12-14 Mike Petrovića Street, Belgrade 11351, Serbia.

[c]Department of Immunology, Institute for Biological Research "Siniša Stanković", National Institute of the Republic of Serbia, University of Belgrade; Bulevar despota Stefana 142, 11060 Belgrade, Serbia

* Correspondence:

Danijela Maksimović-Ivanić

nelamax@ibiss.bg.ac.rs.

Evamarie Hey-Hawkins

hey@rz.uni-leipzig.de

Contents

1. Synthesis and characterisation 3
1.1. Instrumentation 3
1.2. Synthesis of methyl 2-bromoisonicotinate (b) 3
1.3. Synthesis of methyl [2,2'-bipyridine]-4-carboxylate (c) 4
1.4. Synthesis of 4-(hydroxymethyl)-2,2'-bipyridine (d) 5
1.5. Synthesis of 4-fluorophenyl-6-methoxy-2-(4-methoxyphenyl)-benzo[*b*]thophene-3-yl)-methanone (f) 6
1.6. Synthesis of [6-methoxy-2-(4-methoxyphenyl)benzo[*b*]thiophen-3-yl]-[4-(2,2′-bipyridin-4‘-yl-methoxy)phenyl]methanone (bipyraloxifene) (**2**) 7
4. Stability 12
5. In vitro study 13
6. Docking 13
6.1. Oestrogen receptor α 13
6.2. Oestrogen receptor β 15
6.3. Epidermal growth factor receptor 17
6.4. Aryl hydrocarbon receptor 18
6.5. Cannabinoid receptor 2 19
7. References 21

1. Synthesis and characterisation

All reactions were carried out under a dry and oxygen-free nitrogen or argon atmosphere using Schlenk line technique. Tetrahydrofuran (THF) was dried over potassium with benzophenone and kept over molecular sieves 4 Å.[1] Dichloromethane (DCM) was obtained from a solvent purification system and stored over activated molecular sieves 3 Å. Toluene was dried over potassium with benzophenone and kept over molecular sieves 4 Å.[2] Methanol was dried over KOH and stored over molecular sieves 3 Å.[2] Diethyl ether (Et_2O), *n*-hexane and ethyl acetate (EtOAc) were used as purchased. The molecular sieves (3 and 4 Å) were activated under vacuum at 300 °C for 3 h. Silica gel for column chromatography was purchased from Merck (0.035–0.070 mm, 60 Å). Thin layer chromatography (TLC) was conducted on precoated TLC sheets ALUGRAM Xtra SIL G/UV254 (0.20 mm silica gel 60 F254); visualization of the compounds on the plate was achieved with UV light (254 and 366 nm).

1.1. Instrumentation

NMR spectra were recorded at room temperature (25 °C) with a Bruker AVANCE III HD 400 spectrometer. ^{1}H (400.13 MHz) and ^{13}C (100.16 MHz) NMR spectra were referenced to $SiMe_4$ (TMS) as an internal standard. ESI mass spectra were recorded with a Bruker ES-QUIRE 3000 (Benchtop LC Ion trap) mass spectrometer. The FT-IR spectra were obtained with a Nicolette IS5 (ATR) from Thermo Fisher (Waltham, MA, USA) with the scan range 4000–400 cm^{-1}. A Hereaus VARIO EL oven was used to perform elemental analyses.

1.2. Synthesis of methyl 2-bromoisonicotinate (b)

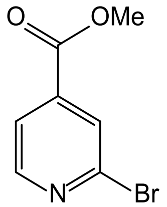

Synthesis of this compound was conducted in accordance with the literature-reported procedure[3] with slight modifications. (3-Dimethylamino-propyl)-ethyl-carbodiimide hydrochloride (EDC·HCl, 1.36 g, 7.23 mmol, 1 eq.) was added at 0 °C under nitrogen to a white suspension of 2-bromoisonicotinic acid (1.46 g, 7.23 mmol, 1 eq.) in 15 mL methanol and 10 mL DCM. The suspension was warmed to room temperature and stirred for 20 h turning into

the transparent solution. The solvents were evaporated under reduced pressure resulting in an oily residue. The residue was transferred to a chromatography column and eluted with *n*-hexane/EtOAc with the gradient 10:1 $\rightarrow$ 5:1 (*n*-hexane:EtOAc). The first fraction was collected and the solvents were evaporated resulting in a liquid residue containing the product. This residue solidified on cooling producing the product as a white powder. 70% yield (1.09 g, 5.06 mmol).

^{1}H NMR (400 MHz, $CDCl_3$), δ [ppm]: 8.53 (d, $^3J_{HH}$ = 5.0 Hz, 1H), 8.05 (t, $^3J_{HH}$ = 0.7 Hz, 1H), 7.81 (dd, $^3J_{HH}$ = 5.0, $^4J_{HH}$ = 1.4 Hz, 1H), 3.97 (s, 3H, -O-CH_3).

1.3. Synthesis of methyl [2,2'-bipyridine]-4-carboxylate (c)

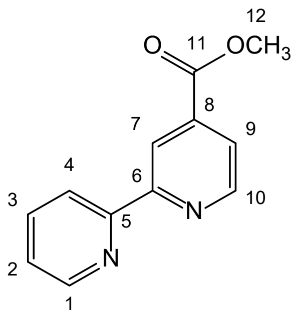

A Stille coupling was conducted to prepare 4-carboxymethyl-2,2’-bipyridine following the procedures described in the literature,[4] however with slight modifications. Compound **b** (1.03 g, 4.78 mmol, 1 eq.) was dissolved in 30 mL dry toluene and an equimolar amount of 2-(tributylstannyl)pyridine (1.56 mL, 4.78 mmol, 1 eq.) was added. The solution was then degassed with N_2 for 30 min before adding 2.5 mol-% of [$Pd(PPh_3)_4$] to the reaction mixture. After stirring at room temperature for 30 min, the mixture was warmed to reflux and stirred with refluxing for 48 h. CsF (0.8 g, 5.30 mmol) was dissolved in distilled water and added to the reaction mixture at room temperature. The phases were separated and the aqueous phase was extracted with toluene (3x20 mL). The combined organic phases were washed with brine (2x20 mL) and distilled water (20 mL). The organic phases were dried with $MgSO_4$, filtered and the solvent was removed under reduced pressure. The crude dark brown residue was further purified using an ISOLERA 4 (silica snap, 25 g, EtOAc/*n*-hexane, 8% $\rightarrow$ 52% EtOAc), resulting in the pure product as a yellow solid (75%, 0.77 g, 3.59 mmol). The NMR spectra are in agreement with those reported in the literature.[4]

^{1}H NMR (400 MHz, $CDCl_3$), δ [ppm]: 8.95 (dd, $^{3}J_{HH}$ = 1.7, $^{4}J_{HH}$ = 0.8 Hz, 1H, 10-C**H**), 8.83 (dd, $^{3}J_{HH}$ = 5.0, $^{4}J_{HH}$ = 0.8 Hz, 1H, 1-C**H**), 8.73 (ddd, $^{3}J_{HH}$ = 4.9, $^{3}J_{HH}$ = 1.9, $^{4}J_{HH}$ = 0.9 Hz, 1H, 7-C**H**), 8.43 (dd, $^{3}J_{HH}$ = 8.0, $^{4}J_{HH}$ 1.3 Hz, 1H, 4-C**H**), 7.89 – 7.86 (m, 1H, 9-C**H**), 7.84 (dd, $^{3}J_{HH}$ = 7.9, $^{4}J_{HH}$ = 1.8 Hz, 1H, 3-CH), 7.35 (ddd, $^{3}J_{HH}$ = 7.5, $^{3}J_{HH}$ = 4.8, $^{4}J_{HH}$ = 1.2 Hz, 1H, 2-CH), 3.99 (s, 3H, 12-CH_3).

1.4. Synthesis of 4-(hydroxymethyl)-2,2'-bipyridine (d)

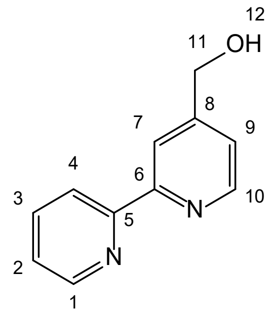

The literature procedure[5] for preparation of alcohol **d** was modified as reported below. Compound **c** (0.40 g, 1.87 mmol, 1 eq.) was dissolved in 30 mL THF under inert conditions. The resulting solution was cooled to 0 °C and $LiBH_4$ (0.12 g, 5.61 mmol, 3 eq.) was added slowly. The reaction mixture was stirred for 1 h at 0 °C, followed by a gradual increase to room temperature, and stirred for 12 h at room temperature. Afterward, the reaction mixture was quenched with a saturated $NaHCO_3$ solution (40 mL). The mixture was transferred to a separation funnel and extracted with DCM (3x30 mL). The combined organic layers were subsequently dried with $MgSO_4$. After filtration, the solvent was removed under reduced pressure. Further purification was accomplished using an ISOLERA 4 with a Silica snap 25 g column and a mobile phase of MeOH/Et_2O (with the gradient 5% $\rightarrow$ 10 % MeOH) to yield the product **5** as a yellow oil (60%, 209 mg, 1.12 mmol).

^{1}H NMR (400 MHz, $CDCl_3$) δ [ppm]: 8.67 (dd, $^{3}J_{HH}$ = 4.9, $^{4}J_{HH}$ = 1.6, 1H, 1-C**H**), 8.63 (d, $^{3}J_{HH}$ = 5.0 Hz, 1H, 10-C**H**), 8.39 (dd, $^{3}J_{HH}$ = 8.0, $^{4}J_{HH}$ = 1.2 Hz, 1H, 4-C**H**), 8.36 (s, 1H, 7-C**H**), 7.83 (td, $^{3}J_{HH}$ = 7.7, $^{3}J_{HH}$ = 1.8 Hz, 1H, 3-C**H**), 7.35-7.31 (m, 2H, 2-C**H**, 9-C**H**), 4.82 (s, 2H, 11-C**H**).

1.5. Synthesis of 4-fluorophenyl-6-methoxy-2-(4-methoxyphenyl)-benzo[*b*]thophene-3-yl)methanone (f)

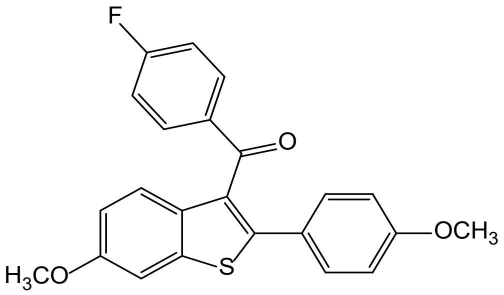

According to the published procedure,[6] 6-methoxy-2-(4-methoxyphenyl)benzo[*b*]thiophene (2.01 g, 7.4 mmol, 1 eq.) was dissolved in 70 mL dry DCM under a nitrogen atmosphere. The solution was cooled to 0 °C with an ice bath. 4-fluorobenzoyl chloride (1 ml, 8.2 mmol, 1.1 eq.) was added followed by $AlCl_3$ (1.41 g, 10.6 mmol, 1.3 eq.), resulting in an immediate color change from white to deep red. The mixture was then gradually warmed to room temperature and left stirring for 12 h. Then the reaction mixture was neutralized with 2M HCl (100 ml), causing the solution to turn yellow. The two phases were separated, and the aqueous phase was extracted with DCM (3x70mL). The combined organic phases were subsequently washed with 2M HCl (1x70 mL) and brine (1x70 mL), followed by water (2x70mL). The organic phases were dried with $MgSO_4$. After filtration, the solvent was evaporated under reduced pressure resulting in a yellow rubbery crude mixture. The product was purified by an ISOLERA4 column (Biotage KP Sil 100g) chromatography with a gradient of 1% $\rightarrow$ 30% of EtOAc in *n*-hexane, resulting in 2.10 g (5.12 mmol, 69%) of pure product **f** as a pale yellow solid after solvent evaporation. The analytical data are in agreement with those reported in the literature.[6]

^{1}H NMR (400 MHz, $CDCl_3$), δ [ppm]: 7.82-7.75 (m, 2H), 7.62 (d, $^3J_{HH}$ = 8.9 Hz, 1H); 7.33 (d, $^3J_{HH}$ = 2.4 Hz, 1H), 7.31-7.28 (m, 2H), 7.00 (dd, $^3J_{HH}$ = 8.9, $^4J_{HH}$ = 2.4 Hz, 1H), 6.92 (t, $^3J_{HH}$ = 8.6 Hz, 2H), 6.74 (m, 2H), 3.89 (s, 3H, -O-CH_3); 3.75 (s, 3H, -O-CH_3).

^{19}F{^{1}H} (400 MHz, $CDCl_3$), δ [ppm]: -104.9.

1.6. Synthesis of [6-methoxy-2-(4-methoxyphenyl)benzo[*b*]thiophen-3-yl]-[4-(2,2'-bipyridin-4'-yl-methoxy)phenyl]methanone (bipyraloxifene) (2)

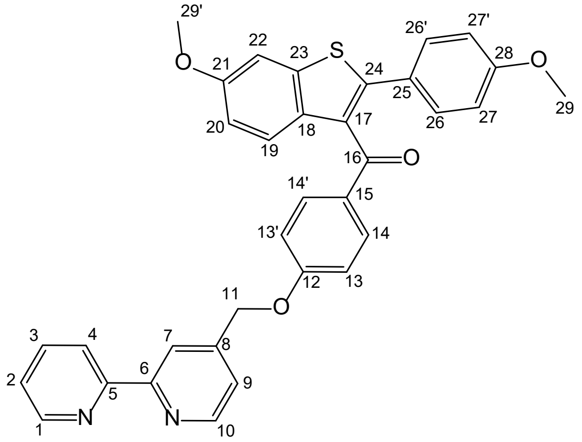

Alcohol ***d*** (200 mg, 1.07 mmol, 1 eq.) was dissolved in 15 mL dry THF and cooled to 0 °C with an ice bath. Then NaH (78.0 mg, 60% w/w in mineral oil, 2 eq.) was added and the reaction mixture was stirred for 75 min with slowly warming to room temperature. Compound **f** (419.5 mg, 1.07 mmol, 1 eq.) was dissolved in 5 mL dry THF; this solution was then added to the reaction mixture dropwise. A color change from yellow to red-orange was observed after 2 h. The reaction mixture was stirred for 12 h, checking the progress with TLC (EtOAc/*n*-hexane 1:1) and NMR spectroscopy. After this time, the reaction mixture was quenched by adding distilled water (20 mL). Ethyl acetate was added, and the organic and aqueous phase were separated. The aqueous phase was extracted with ethyl acetate (3x10 mL) and the combined organic phases were washed once with brine (10 mL) and water (10 mL). The organic phase was dried with $MgSO_4$, filtered and the solvent was evaporated under reduced pressure. The crude product was purified using an ISOLERA 4 (SNAP Ultra KP-Sil 50 g, EtOAc/*n*-hexane, 10% $\rightarrow$ 80% of EtOAc) giving 418 mg (0.75 mmol, 70%) of bipyraloxifene (**2**) as a pale yellow powder.

M = 558.16 g mol^{-1}. .**^{1}H NMR** (400 MHz, $CDCl_3$), δ [ppm]: 8.69 (t, $^3J_{HH}$ = 4.2 Hz, 2H, 1-C**H**, 10-C**H**), 8.42 (d, $^3J_{HH}$ = 6.9 Hz, 2H, 4-C**H**, 7-C**H**), 7.85 (td, $^3J_{HH}$ = 7.7 Hz, $^3J_{HH}$ = 1.8 Hz, 1H, 3-C**H**), 7.79 (d, $^3J_{HH}$ = 8.9 Hz, 2H, 14,14'-C**H**), 7.55 (d, $^3J_{HH}$ = 9.0 Hz, 1H, 19-C**H**), 7.36-7.30 (m, 5H, 22-C**H**, 26,26'-C**H**, 2-C**H**, 9-C**H**), 6.96 (dd, $^3J_{HH}$ = 8.9, $^4J_{HH}$ = 2.4 Hz, 1H, 20-C**H**), 6.85 (d, $^3J_{HH}$ = 8.9 Hz, 2H, 13,13'-C**H**), 6.75 (d, $^3J_{HH}$ = 8.7 Hz, 2H, 27,27'-C**H**), 5.17 (s, 2H, 11-C**H$_2$**), 3.89 (s, 3H, 29'-C**H$_3$**), 3.76 (s, 3H, 29-C**H$_3$**). **^{13}C{^{1}H}** (101 MHz, $CDCl_3$), δ [ppm]: 193.1 (16-**C**), 162.2 (12-**C**), 159.8 (28-**C**), 157.7 (21-**C**), 156.5 (6-**C**), 155.7 (5-**C**), 149.6 (10-**C**H), 149.2 (1-**C**H), 146.3 (8-**C**), 142.9 (24-**C**), 140.1 (18-**C**), 137.1 (3-**C**H), 133.9 (23-**C**), 132.4 (14,14'-**C**H), 131.1 (15-**C**), 130.4 (17-**C**), 130.3 (26,26'-**C**H), 126.0 (25-**C**), 124.1 (19-**C**H), 124.0 (2-**C**H), 121.4 (4-**C**H) , 121.3 (9-**C**H), 118.8 (7-

CH), 114.8 (20-**C**H), 114.4 (13,13′-**C**H), 114.1 (27,27′-**C**H), 104.5 (22-**C**H), 68.4 (11-**C**H_2), 55.6 (29′-**C**H_3), 55.3 (29-**C**H_3). **UV-vis** in DCM, λ_{max}, nm (ε, $cm^{-1}M^{-1}$, C = 5.6 μM, l = 1 cm): 285 ($1.79{\cdot}10^5$), 368 ($0.20{\cdot}10^5$). **Elemental analysis:** $C_{34}H_{26}N_2O_4S$ calc. C, 73.10; H, 4.69; N, 5.01, found C, 72.81; H, 4.59; N, 4.97 (%).

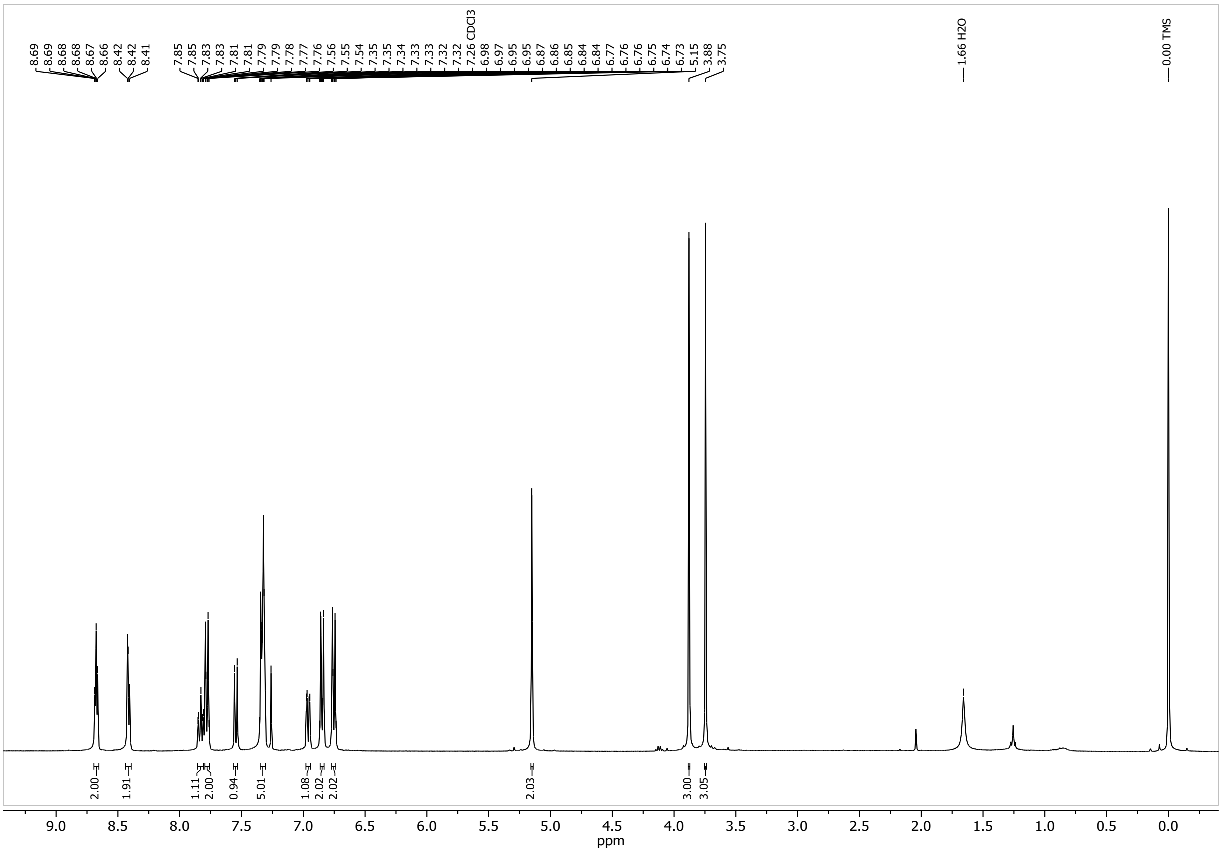

Figure S1. ^1H NMR spectrum of **2** in $CDCl_3$.

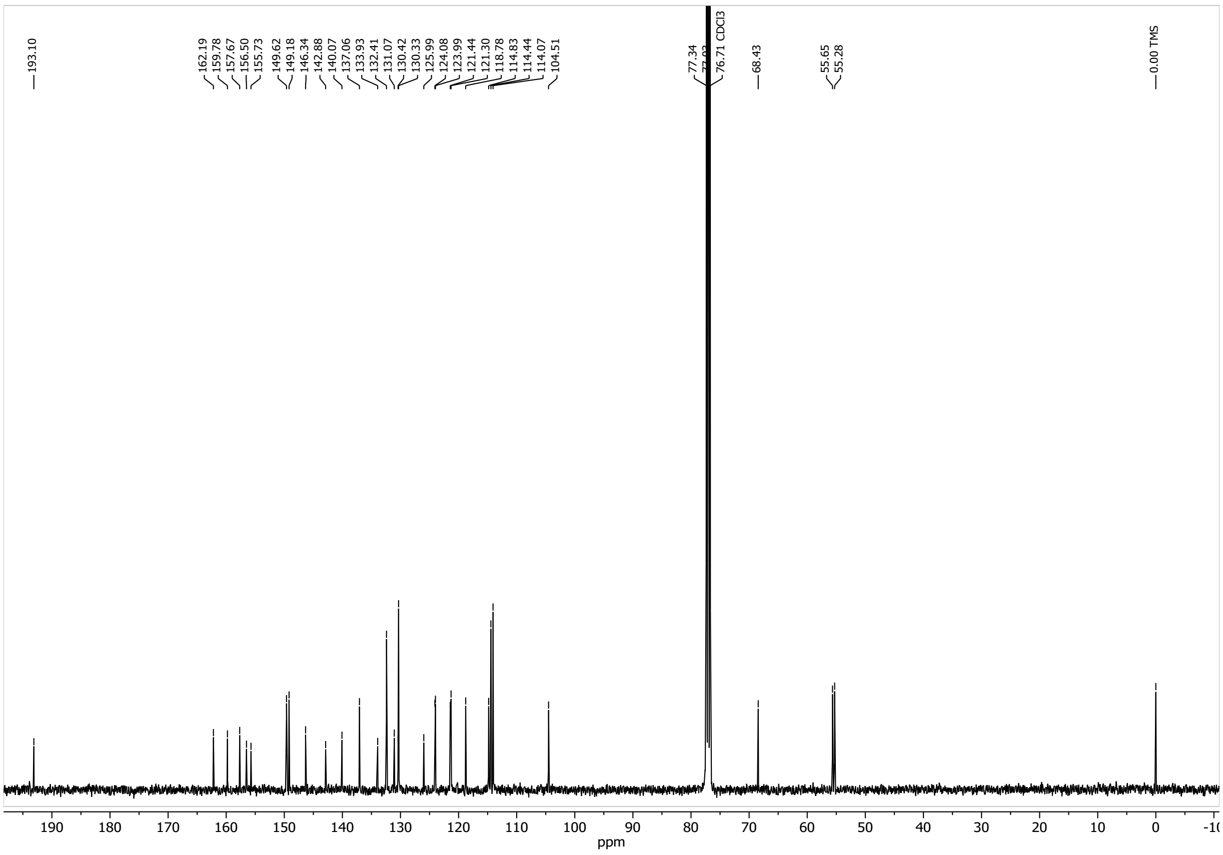

Figure S2. $^{13}C\{^{1}H\}$ NMR spectrum of **2** in $CDCl_3$.

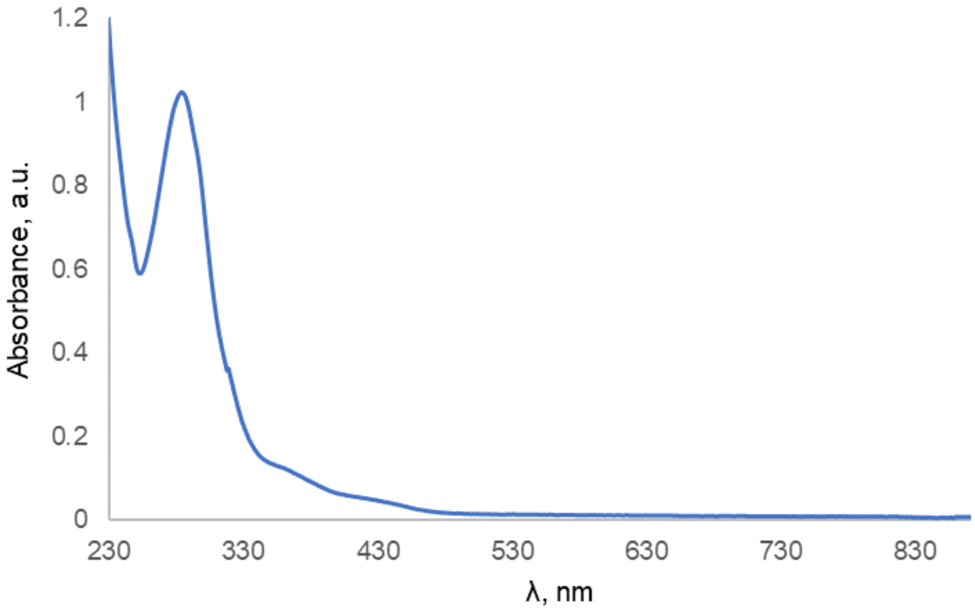

Figure S3. UV-vis spectrum of **2** recorded in DCM.

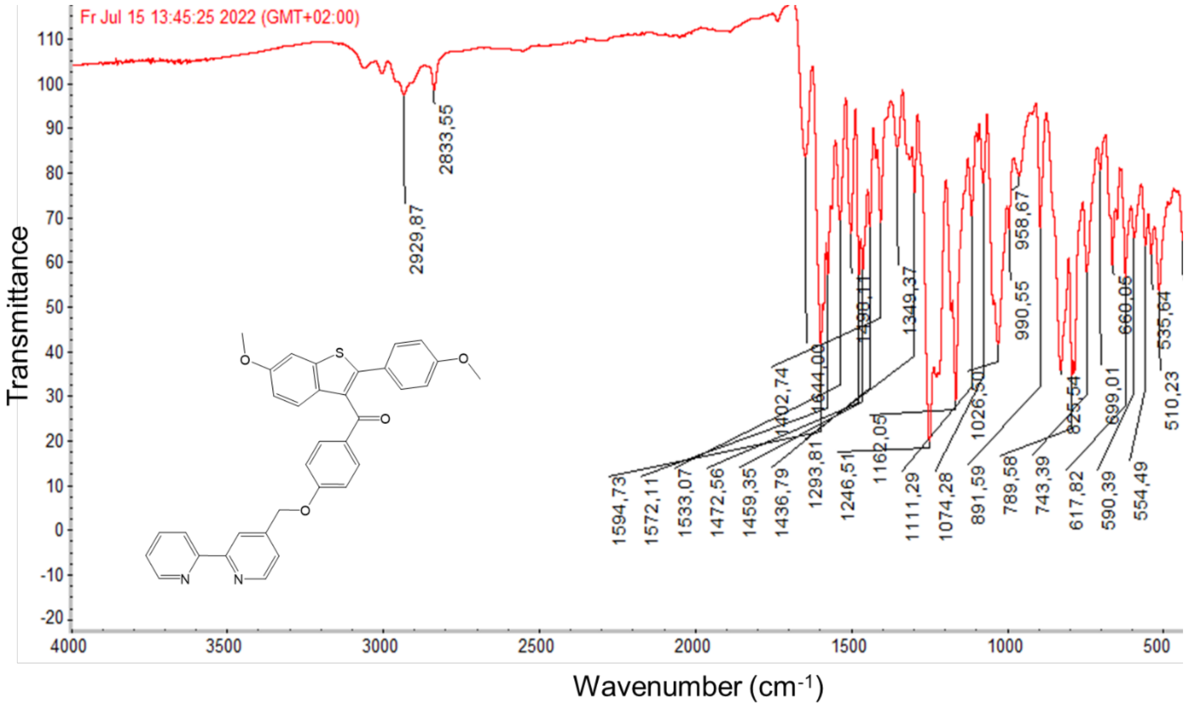

Figure S4. Infrared spectrum of **2**: 3000-2834 (w, C_{alk}–H stretching of -O-CH_3 and -O-CH_2-), 1644 (w, C-$C_{aromatic}$ stretching of 2,2'-bipy), 1595 (s, C-$C_{aromatic}$ vibration of benzothiophene and C=O stretch of keto group), 1572 (w, C-$C_{aromatic}$ vibration of benzothiophene), 1533 (s, C_{arom}–H in-plane bending of 2,2'-bipy), 1498 (w, C_{arom}–H in-plane bending), 1473 (w, C_{alk}–H bending of -O-CH_3), 1459 (w, C-$C_{aromatic}$ vibration and C_{arom}-H in-plane bending), 1437 (w, C_{alk}–H bending of -O-CH_2-), 1384 (w, C-$C_{aromatic}$ vibration and C_{arom}–H in-plane bending of benzothiophene), 1349 (w, C-$C_{aromatic}$ vibration of 2,2´-bipy), 1294 (w, C_{alk}-H bending of -CH_2-), 1247(s, C_{arom}-O of -Ph-O-CH_3 and C_{arom}-H in-plane bending of phenyl rings), 1162 (s, -H_2C-O- stretching and C-H in-plane bending), 1111 (w, C-$C_{aromatic}$ vibration of 2,2´-bipy and phenyl), 1074 (w, C-$C_{aromatic}$ vibration of 2,2'-bipy), 1027 (s, Calk–O), 991 (w, C-$C_{aromatic}$ vibration of 2,2´-bipy), 959 (w, $C_{aromatic}$-H out-of-plane bending of 2,2´-bipy), 892 (m, $C_{aromatic}$-H out-of-plane bending of phenyl), 826 (m, $C_{aromatic}$-H out-of-plane bending of phenyl), 790 (m, $C_{aromatic}$-H out-of-plane bending and $C_{aromatic}$-C_{ketone} stretching) 743 (m, C-$C_{aromatic}$ out-of-plane bending), 724 (w, $C_{aromatic}$-H out-of-plane bending of phenyl), 699 (w, C-$C_{aromatic}$ vibrations of phenyl and 2,2'-bipy), 660 (w, $C_{aromatic}$-H out-of-plane bending of phenyl and 2,2'-bipy), 618 (w, C-$C_{aromatic}$ out-of-plane bending of phenyl and 2,2'-bipy), 590 (w, C-$C_{aromatic}$ vibrations of 2,2'-bipy), 555 (w, $C_{aromatic}$-H out-of-plane bending of phenyl), 536 (w, $C_{aromatic}$-H out-of-plane bending of phenyl and 2,2'-bipy), 510 (w, C-$C_{aromatic}$ vibrations of benzothiophene and C-S vibration). Identification of the bond vibrations was based on calculated frequencies (Figure S5).

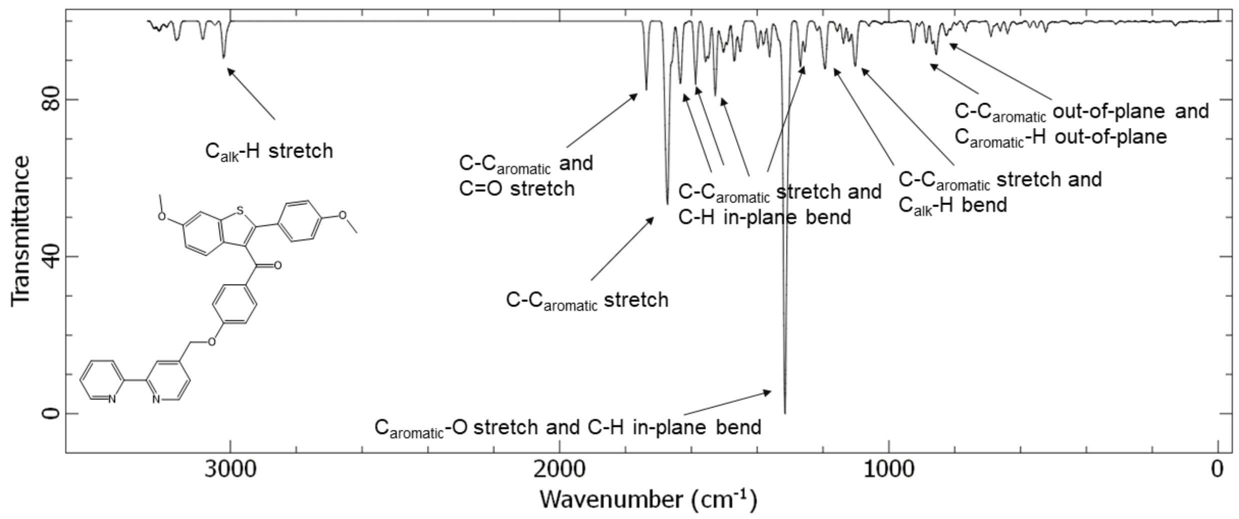

Figure S5. Calculated IR spectrum of ligand **2** using DFT at PBE0 D3BJ/def2-TZVPP level of theory.

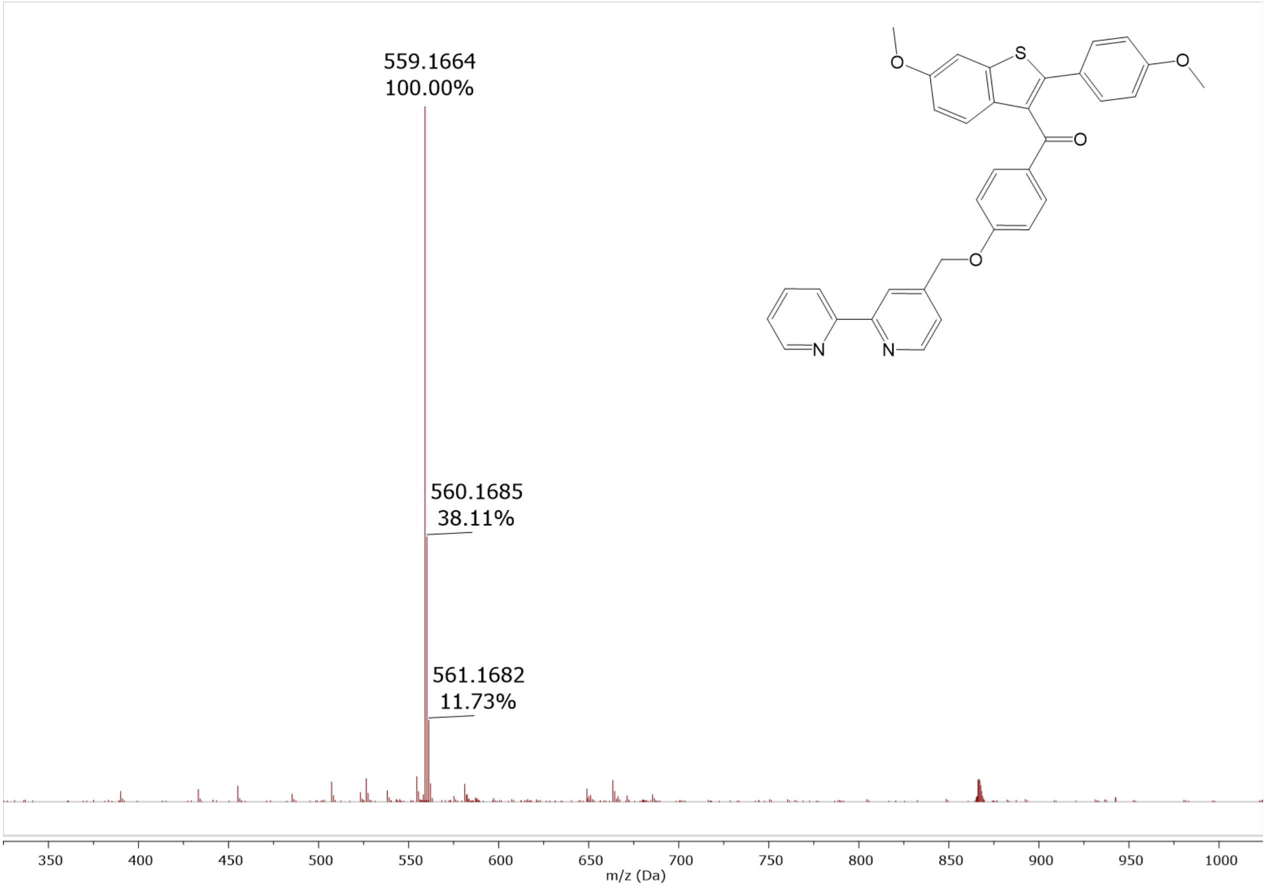

Figure S6. HR-ESI-MS (positive mode, acetonitrile) of **2**, M = 558.1613: m/z $[M+H]^{+}$= 559.1686 (calc.), 559.1664 (found).

4. Stability

For the in vitro tests, stock solutions of compounds **1** and **2** in DMSO were prepared and stored at +4 °C. To assure the stability of compound **2**, ^{1}H NMR spectra were recorded in water-containing DMSO-d_6 in air and kept at +4 °C between measurements. Compound **2** is stable in DMSO solution for at least two months.

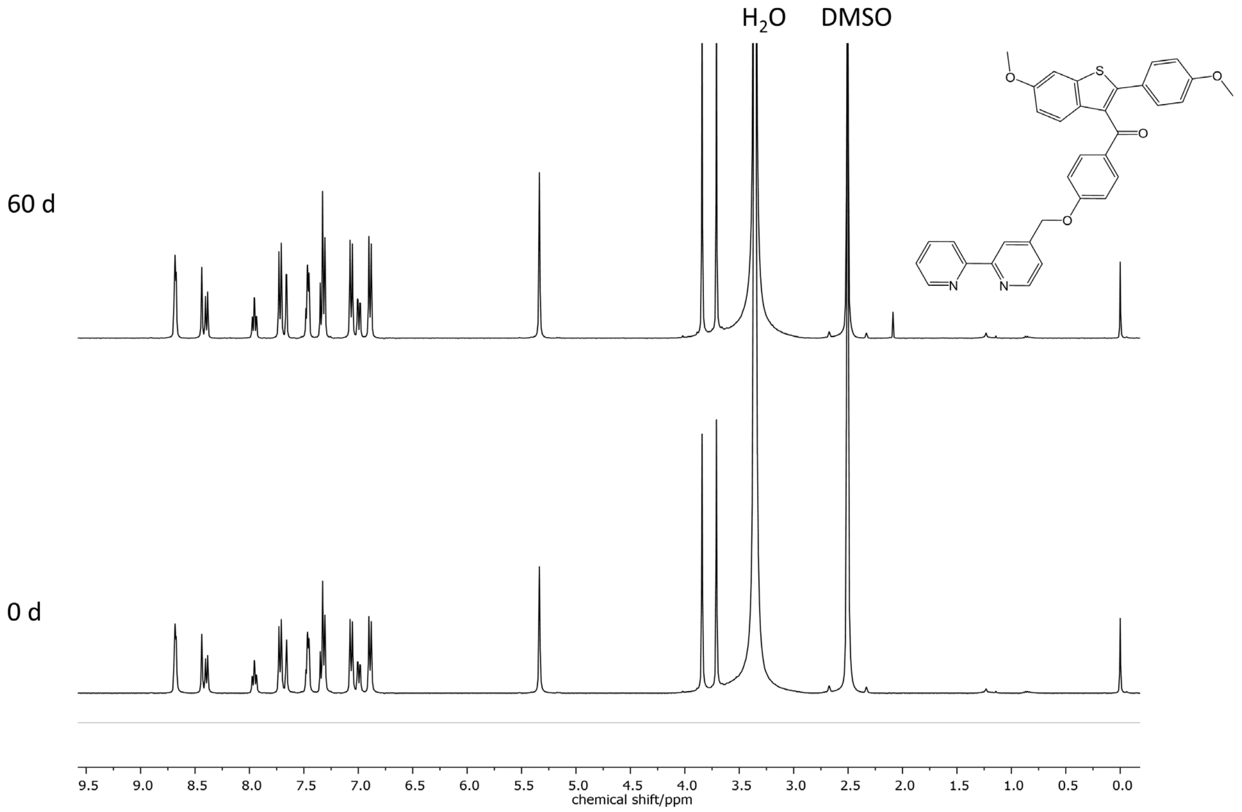

Figure S7. ^{1}H-NMR spectra of bipyraloxifene (**2**) in water-containing DMSO-d_6 over 60 days at room temperature, but stored at +4 °C between the measurements. No changes could be observed.

5. In vitro study

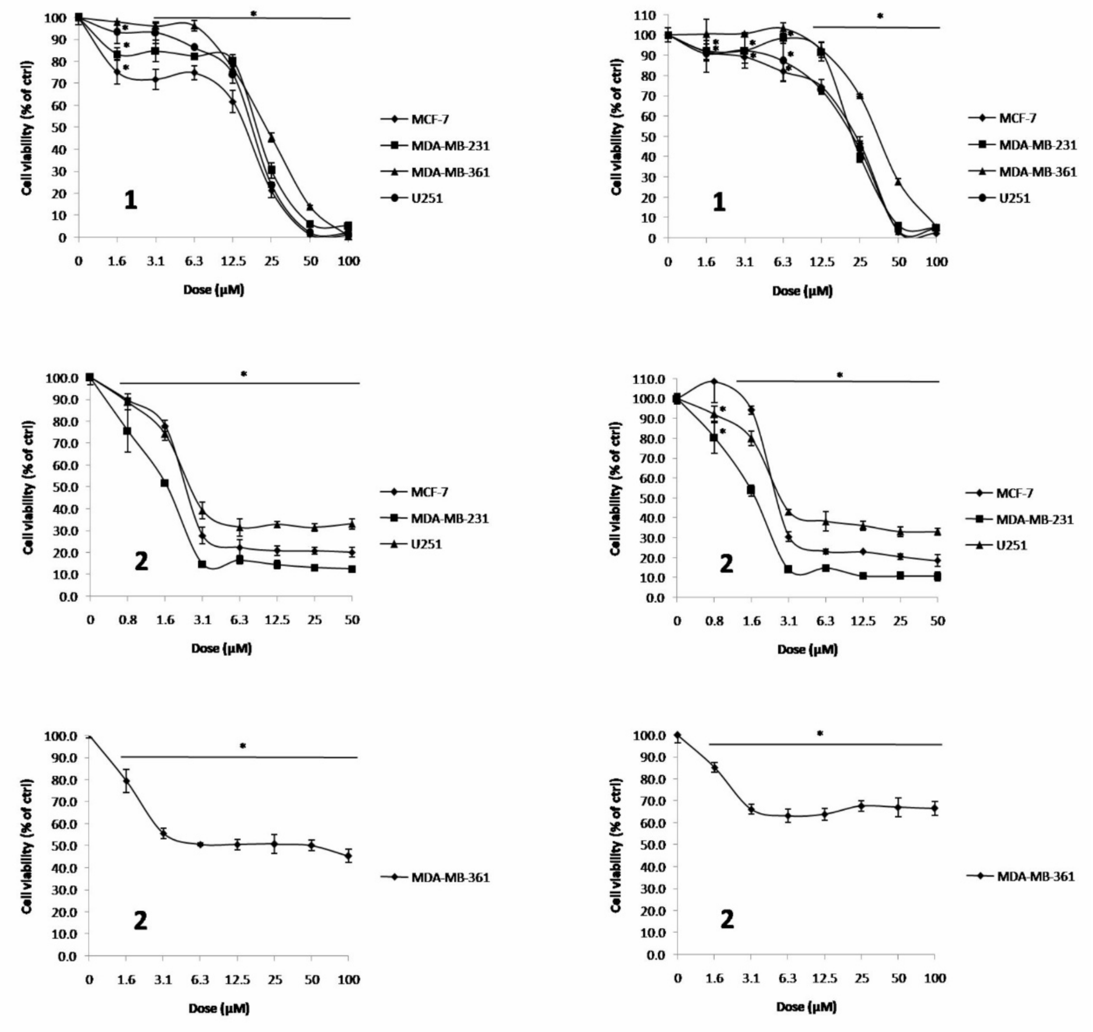

Figure S8. The impact of bipyraloxifene (**2**) on the viability of tumor cell lines. Cells were exposed to raloxifene (**1**) or bipyraloxifene (**2**). MTT (left panel) and CV (right panel) assays were done after 72 h. The data are presented as the mean ± SD of one representative experiment out of three independent and expressed as a percentage of untreated cells that was arbitrarily set as 100%. $^{*}p < 0.05$

6. Docking

6.1. Oestrogen receptor α

The molecular docking was performed with the AutoDockTools4 software[7] using the Lamarckian Genetic Algorithm[8]. The water molecules were eliminated and the non-polar hydrogen atoms were merged. The docking area was limited by the constructed grid box of the size 54 x 62 x 54 centred at 42.912, 23.687, 77.071 of x,y,z-coordinates (based on the position of the LBD). The following parameters were used in the docking: number of hybrid GA-LS runs: 100; population size: 150; maximum number of energy evaluations: 2,500,000,

maximum number of top individuals to survive to next generation: 1; rate of gene mutation: 0.02; rate of crossover: 0.8; Mean of Cauchy distribution for gene mutation: 0.0; variance of Cauchy distribution for gene mutation: 1.0 (Figure S9).

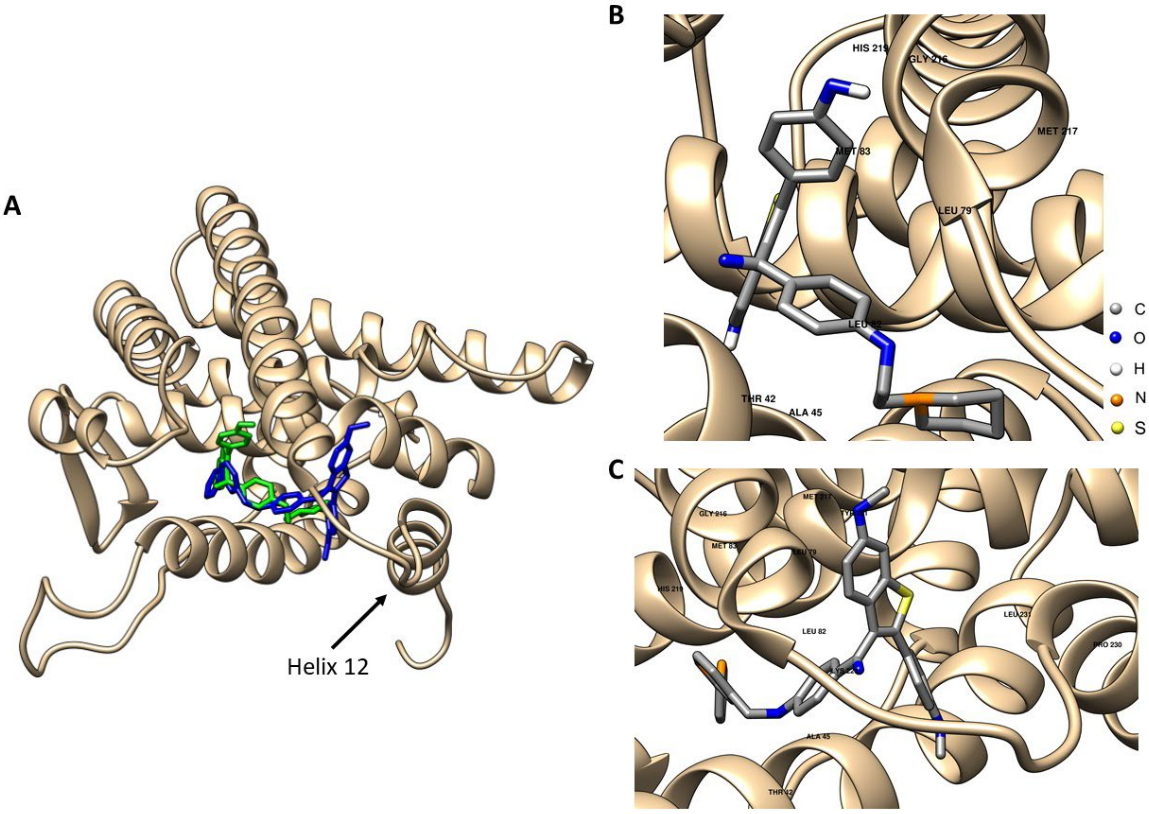

Figure S9. *In silico* investigation of the binding modes of compounds raloxifene (**1**) and bipyraloxifene (**2**) based on docking in oestrogen receptor α (the structure with PDB code 1ERR[9] was used as the starting point for docking). The highest ranked docked positions of compounds **1** (green, **A**) and **2** (blue, **A**) are shown compared to each other. The highest ranked position of **1** (B) and **2** (C) are shown in the ligand-binding pocket of the receptor together with the labeled amino acid residues important for the interactions.

To aid the understanding of the binding abilities of bipyraloxifene to ERα, we conducted docking studies to estimate the binding mode and binding energies. The crystal structure of ERα with raloxifene inside the ligand-binding pocket of the receptor is available in the Protein Data Bank (PDB) under the code 1ERR[9] and was used as the starting point for the docking studies. Raloxifene was docked in the position of its original crystal structure, exhibiting a calculated binding energy of -12.5 kcal/mol. Interestingly, the incorporation of the 2,2′-bipy moiety decreased the affinity to -11.0 kcal/mol for bipyraloxifene and reoriented the position of the benzo[*b*]thiophene moiety in the opposite direction compared to raloxifene

(see Figure S9, A). Although the set of amino acid residues of ERα participating in the interactions remained similar compared to the original raloxifene structure (see Figure S9, B), the 2,2'-bipy unit is now oriented inside the binding pocket, pushing the benzo[*b*]thiophene moiety towards helix 12 (see Figure S9, C). This helix plays an essential role in activation function 2, responsible for the interaction of ERα with other protein activators. Additionally, it is essential to note that the binding energy of structure **2** is comparable to the binding energy of another SERM, 4-hydroxytamoxifen, to ERα, which was calculated and reported by us earlier (-10.8 kcal/mol)[10]. Therefore, this modelling indicates the possibility that bipyraloxifene binds to ERα with an affinity that is considerable for SERMs.

6.2. Oestrogen receptor β

The similar software and parameters were applied for docking into oestrogen receptor β (ERβ). However, the docking area was limited by the constructed grid box of the size 56 x 54 x 56 centred at 20.123 29.747 9.322 of x,y,z-coordinates for raloxifene or 60 x 54 x 54 centred at 22.601 30.964 11.333 for bipyraloxifene. The position of the grid box was selected based on the position of the original drug raloxifene[11] in the binding pocket of ERβ available at PDB under the code 1QKN[11]. Bipyraloxifene was docked into the coordinates of raloxifene (Figure S10).

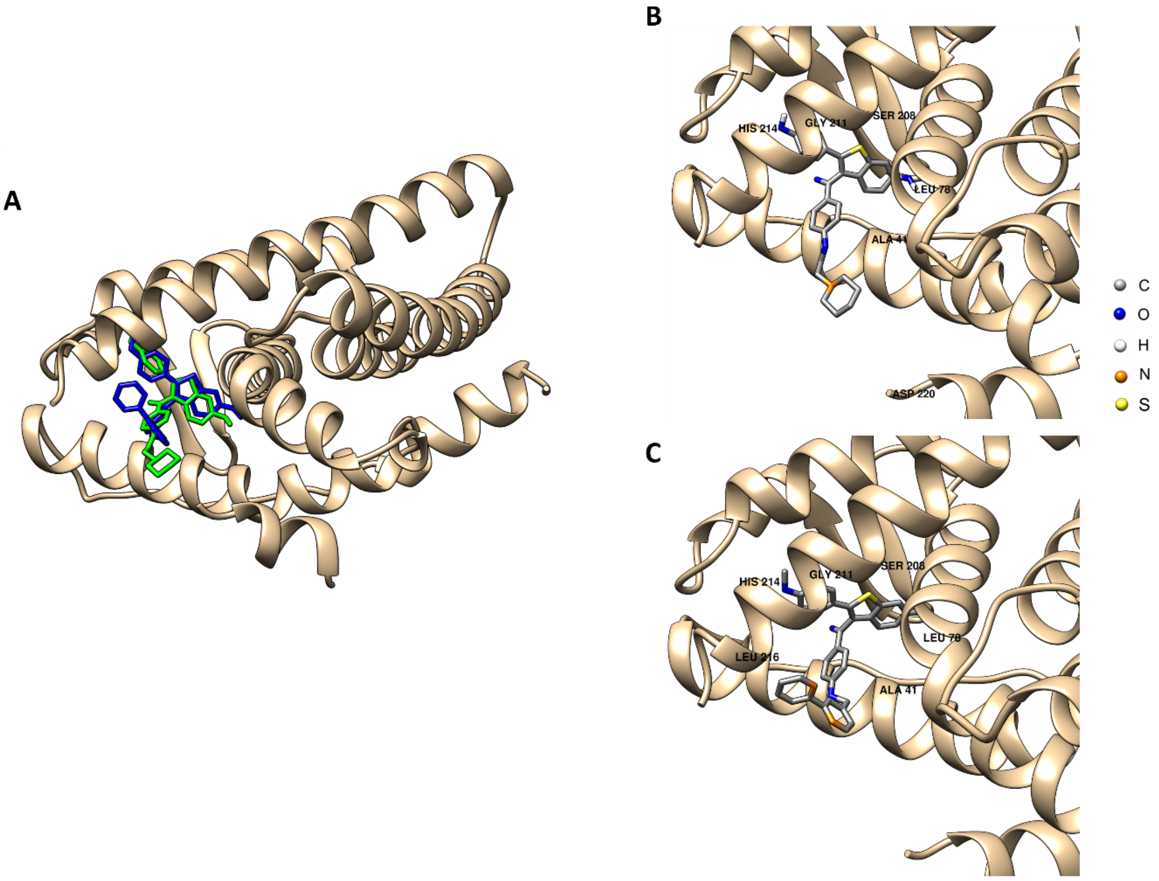

Figure S10. *In silico* investigation of the binding modes of compounds raloxifene (**1**) and bipyraloxifene (**2**) based on docking in oestrogen receptor β (the structure with PDB code 1QKN[11] was used as the starting point for docking). The highest ranked docked position of compound **2** (blue, **A**) is shown in comparison to the crystal structure of raloxifene in the binding pocket. The crystal structure of **1** (B) and the highest ranked position of **2** (C) are shown together with the labeled amino acid residues important for the interactions with the ligands in the binding pocket.

Earlier it was assumed that endocytosis of EGFR which is responsible for suppression of TNBC tumour (as it was shown for the MDA-MB-231 cell line) might be possibly related to the inhibition of ERβ upon binding to raloxifene as ERβ interacts with EGFR.[12] Raloxifene as a SERM exhibits high affinity to both ERα and ERβ.[9,11] In order to investigate the binding mode of the synthesised drug **2** we conducted docking of this compound in the ligand-binding domain of the receptor. Noteworthy, incorporation of the 2,2'-bipy moiety in the raloxifene structure only insignificantly decreases the binding affinity (-12.6 kcal/mol for raloxifene vs -11.9 kcal/mol for bipyraloxifene). The highest ranked docked position of bipyraloxifene demonstrated the similar binding mode and interactions with the same amino acid residues. The structure of **2** aligned in the same manner as raloxifene (Figure S10, A, B and C).

6.3. Epidermal growth factor receptor

The similar software and parameters were applied for docking into epidermal growth factor receptor (EGFR). However, the docking area was limited by the constructed grid box of the size 44 x 48 x 52 centred at 50.617 -3.445 22.821 of x,y,z-coordinates for raloxifene or 60 x 48 x 58 centred at 48.09 -2.302 24.048 for bipyraloxifene. The position of the grid box was selected based on the position of the macrocyclic inhibitor M19 (4-(5-chloro-4-fluoro-2-hydroxyanilino)-7-methoxyquinazolin-6-ol, not shown here)[13] in the binding pocket of EGFR available at PDB under the code 7U9A[13]. M19 was removed from the binding pocket and raloxifene or bipyraloxifene was inserted in the binding pocket of the receptor (Figure S11).

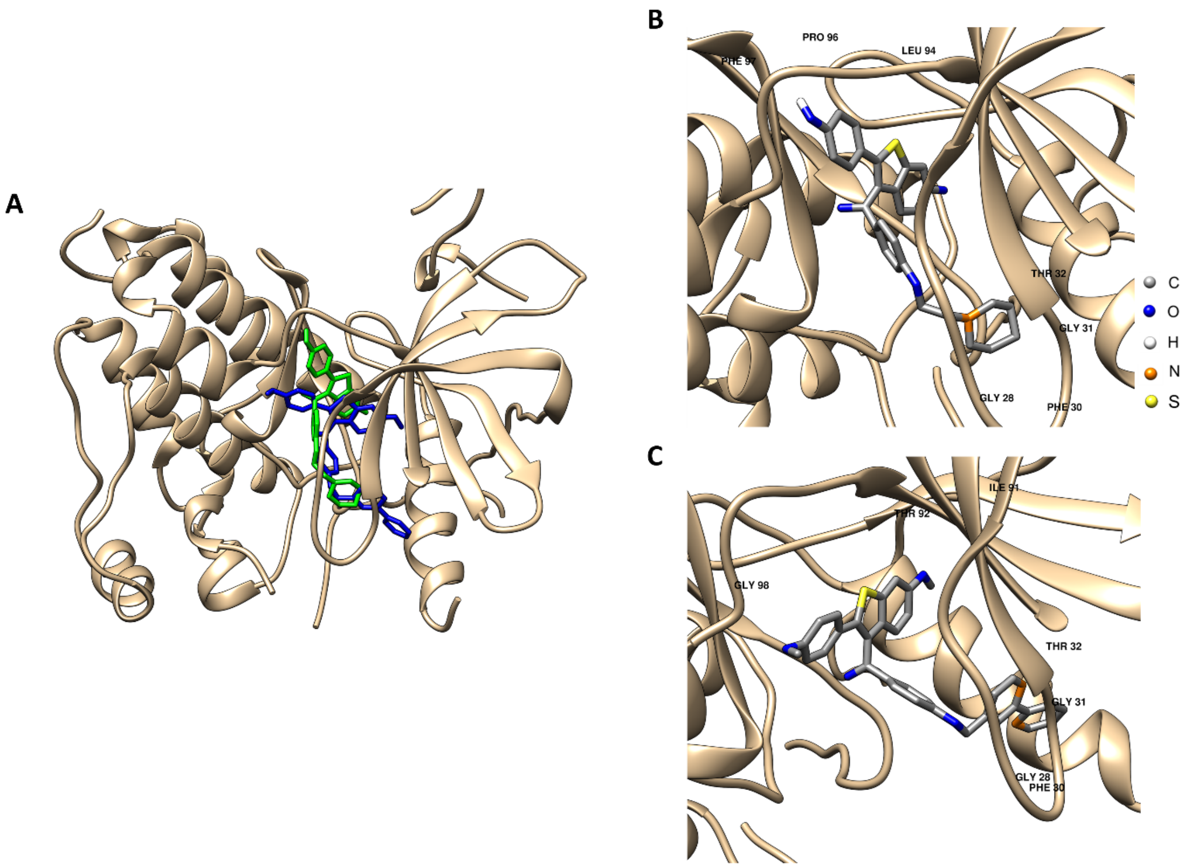

Figure S11. *In silico* investigation of the binding modes of compounds raloxifene (**1**) and bipyraloxifene (**2**) based on docking in the epidermal growth factor receptor (the structure with PDB code 7U9A[13] was used as the starting point for docking). The highest ranked docked positions of compounds **1** (green, **A**) and **2** (blue, **A**) are shown compared to each other. The highest ranked position of **1** (B) and **2** (C) are shown together with the labeled amino acid residues important for the interactions with the ligands in the binding pocket.

As it was shown earlier raloxifene reduces the tumour progression decreasing the expression of EGFR.[12] We selected this receptor to investigate the binding mode of our

bipyraloxifene towards this receptor and compare the binding energies of the synthetic agonist M19, the original drug raloxifene and bipyraloxifene. Although the crystal structure of this receptor was obtained together with M19111 in the binding pocket of the receptor the binding energy made up -7.8 kcal/mol. This value indicates the lowest affinity to this receptor in the row of the ligands M19, raloxifene (-9.0 kcal/mol) and bipyraloxifene (-9.0 kcal/mol), where raloxifene and bipyraloxifene demonstrated higher affinity. The highest ranked positions of compounds **1** and **2** align almost in the similar way interacting almost with the similar amino acid residues (Figure S11, B and C).

6.4. Aryl hydrocarbon receptor

The similar software and parameters were applied for docking into the aryl hydrocarbon receptor (AhR). However, the docking area was limited by the constructed grid box of the size 54 x 62 x 54 centred at -20.329 3.497 8.445 of x,y,z-coordinates. The position of the grid box was selected based on the position of the antagonist alpha-naphthoflavone (αNF) in the binding pocket of drosophila AHR available at PDB under the code 7VNH[14]. αNF was removed from the binding pocket and raloxifene or bipyraloxifene was inserted in the binding pocket of the receptor (Figure S12).

Interestingly, raloxifene has been found to induce apoptosis in ER- cell lines. This mechanism is based on its interaction with the AhR, where raloxifene acts as a ligand for the AhR.[15,16] In order to elucidate this observation and explore potential oestrogen receptor-independent mechanism of bipyraloxifene, we opted to conduct docking studies of raloxifene and bipyraloxifene into the AHR.

Recent studies have established the ligand binding mechanism of this receptor using Drosophila AHR, for which the crystal structure with the antagonist αNF was reported.[14] Initially, we assessed the binding energy of αNF in the binding pocket to provide a benchmark value (-10.5 kcal/mol). Raloxifene demonstrated lower binding affinity (with a binding energy of -7.7 kcal/mol), although its top-ranked positions were found within the hydrophobic pocket of the receptor, suggesting a potential capability to occupy the position of the initial antagonist αNF (Figure S12, B).

The top-ranked position of bipyraloxifene indicated a decrease in its binding affinity to AHR (with a binding energy of -4.6 kcal/mol), wherein the incorporation of the 2,2'-bipy unit caused a shift in the position of bipyraloxifene away from the hydrophobic pocket of the receptor (Figure S12 A and C). However, the interaction of bipyraloxifene with AhR could not

be entirely excluded, as the binding energy remained negative, potentially indicating a mode of action similar to that of raloxifene against TNBC.

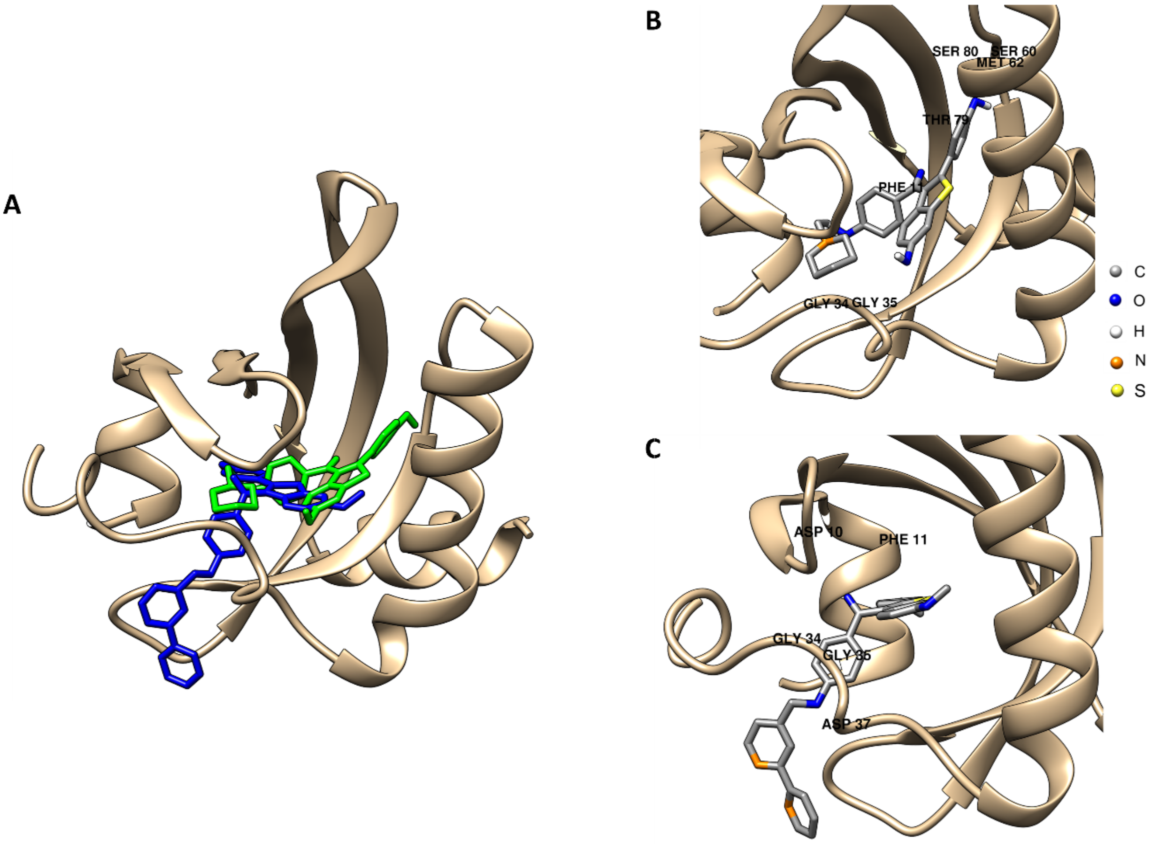

Figure S12. *In silico* investigation of the binding modes of compounds raloxifene (**1**) and bipyraloxifene (**2**) based on docking in aryl hydrocarbon receptor (the structure with PDB code 7VNH[14] was used as the starting point for docking). The highest ranked docked positions of compounds **1** (green, **A**) and **2** (blue, **A**) are shown compared to each other. The highest ranked position of **1** (B) and **2** (C) are shown together with the labeled amino acid residues important for the interactions.

6.5. Cannabinoid receptor 2

The similar software and parameters were applied for docking into cannabinoid receptor 2 (CB2R). However, the docking area was limited by the constructed grid box of the size 52 x 48 x 52 centred at 9.723 0.995 -46.984 of x,y,z-coordinates for raloxifene or 52 x 48 x 52 centred at 11.11 2.212 -43.946 for bipyraloxifene. The position of the grid box was selected based on the position of the synthetic inhibitor AM12033 (7-[(6aR,9R,10aR)-1-hydroxy-9-(hydroxymethyl)-6,6-dimethyl-6a,7,8,9,10,10a-hexahydro-6H-benzo[*c*]chromen-3-yl]-7-methyloctanenitrile)[17] in the binding pocket of CBR2 available at PDB under the code

6KPC[17]. AM12033 was removed from the binding pocket and raloxifene or bipyraloxifene was inserted in the binding pocket of the receptor (Figure S13).

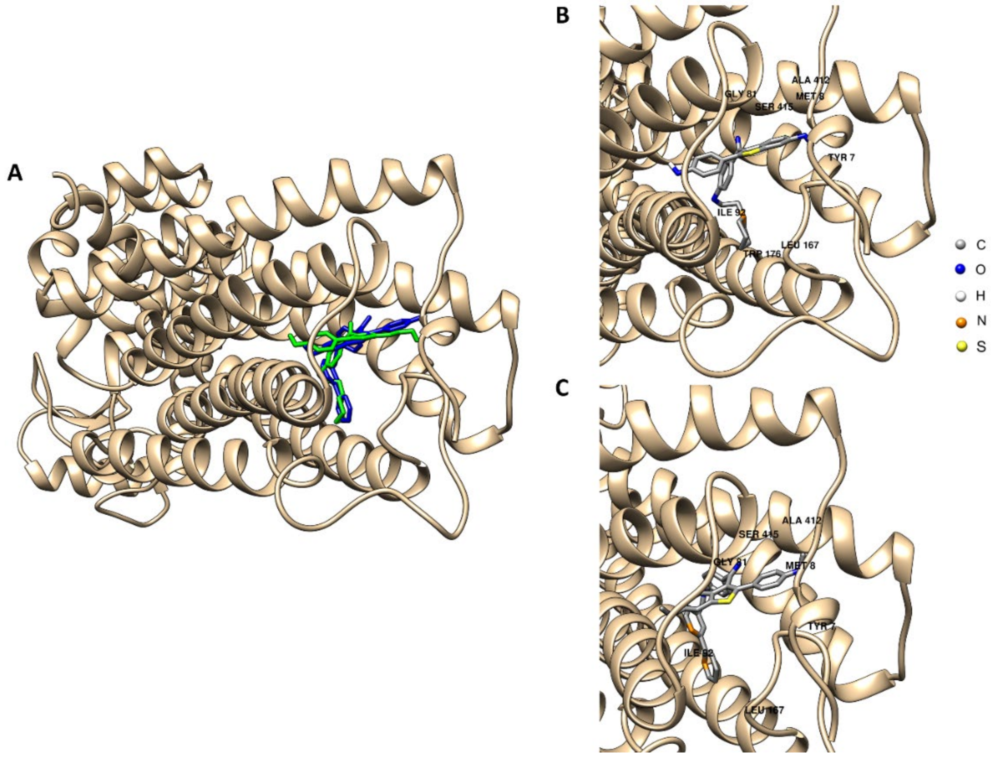

Figure S13. *In silico* investigation of the binding modes of compounds raloxifene (**1**) and bipyraloxifene (**2**) based on docking in cannabinoid receptor 2 (the structure with PDB code 6KPC[17] was used as the starting point for docking). The highest ranked docked positions of compounds **1** (green, **A**) and **2** (blue, **A**) are shown compared to each other. The highest ranked position of **1** (B) and **2** (C) are shown together with the labeled amino acid residues important for the interactions with the ligands in the binding pocket.

CB2R plays a fundamental role in the tumour genesis and might be an interesting target in the treatment of TNBC as well.[18] It was also reported that CB2R is a molecular target for tamoxifen and its metabolite 4-hydroxytamoxifen.[19] We decided to consider the binding affinity of bipyraloxifene to this receptor. The crystal structure of this receptor was available at PDB crystalised together with the synthetic inhibitor AM12033.[17] The binding energies were analysed in comparison to the binding energy of AM12033 (-13.1 kcal/mol). The binding affinity of raloxifene was insignificantly higher than the one observed for AM12033 (-13.3 kcal/mol), while bipyraloxifene showed the lowest binding ability to CB2R in this row with the binding energy -11.8 kcal/mol. However, bipyraloxifene's binding energy is still comparable to the energies of raloxifene and AM12033 indicating the possibility of CB2R as a target for

compound **2**. The docked structures of raloxifene and bipyraloxifene exhibit a similar alignment in the binding pocket of the receptor (Figure S13, A, B and C).

7. References

1 A. B. C. Simas, V. L. P. Pereira, C. B. Barreto Jr., D. L. de Sales and L. L. de Carvalho, *Quím. Nova*, 2009, **32**, 2473–2475.

2 D. B. G. Williams and M. Lawton, *J. Org. Chem.*, 2010, **75**, 8351–8354.

3 Y. Miyake, Y. Itoh, Y. Suzuma, H. Kodama, T. Kurohara, Y. Yamashita, R. Narozny, Y. Hanatani, S. Uchida and T. Suzuki, *ACS Catal.*, 2020, **10**, 5383–5392.

4 H.-C. Lin, H. Kim, S. Barlow, J. M. Hales, J. W. Perry and S. R. Marder, *Chem. Commun.*, 2010, **47**, 782–784.

5 Y. Kobayashi, M. Hoshino, T. Kameda, K. Kobayashi, K. Akaji, S. Inuki, H. Ohno and S. Oishi, *Inorg. Chem.*, 2018, **57**, 5475–5485.

6 S. M. Ervin, R. P. Hanley, L. Lim, W. G. Walton, K. H. Pearce, A. P. Bhatt, L. I. James and M. R. Redinbo, *ACS Chem. Biol.*, 2019, **14**, 2737–2744.

7 G. M. Morris, R. Huey, W. Lindstrom, M. F. Sanner, R. K. Belew, D. S. Goodsell and A. J. Olson, *J. Comput. Chem.*, 2009, **30**, 2785–2791.

8 G. M. Morris, D. S. Goodsell, R. S. Halliday, R. Huey, W. E. Hart, R. K. Belew and A. J. Olson, *J. Comput. Chem.*, 1998, **19**, 1639–1662.

9 A. M. Brzozowski, A. C. W. Pike, Z. Dauter, R. E. Hubbard, T. Bonn, O. Engström, L. Öhman, G. L. Greene, J.-Å. Gustafsson and M. Carlquist, *Nature*, 1997, **389**, 753–758.

10 A. Kazimir, B. Schwarze, P. Lönnecke, S. Jelača, S. Mijatović, D. Maksimović-Ivanić and E. Hey-Hawkins, *Pharmaceutics*, 2023, **15**, 682.

11 A. C. W. Pike, A. M. Brzozowski, R. E. Hubbard, T. Bonn, A.-G. Thorsell, O. Engström, J. Ljunggren, J.-Å. Gustafsson and M. Carlquist, *EMBO J.*, 1999, **18**, 4608–4618.

12 S. Taurin, K. M. Allen, M. J. Scandlyn and R. J. Rosengren, *Int. J. Oncol.*, 2013, **43**, 785–792.

13 J. A. Amrhein, T. S. Beyett, W. W. Feng, A. Krämer, J. Weckesser, I. K. Schaeffner, J. K. Rana, P. A. Jänne, M. J. Eck, S. Knapp and T. Hanke, *J. Med. Chem.*, 2022, **65**, 15679–15697.

14 S. Dai, L. Qu, J. Li, Y. Zhang, L. Jiang, H. Wei, M. Guo, X. Chen and Y. Chen, *Nat. Commun.*, 2022, **13**, 6234.

15 E. F. O'Donnell, D. C. Koch, W. H. Bisson, H. S. Jang and S. K. Kolluri, *Cell Death Dis.*, 2014, **5**, e1038–e1038.

16 H. S. Jang, M. Pearce, E. F. O'Donnell, B. D. Nguyen, L. Truong, M. J. Mueller, W. H. Bisson, N. I. Kerkvliet, R. L. Tanguay and S. K. Kolluri, *Biology*, 2017, **6**, 41.

17 T. Hua, X. Li, L. Wu, C. Iliopoulos-Tsoutsouvas, Y. Wang, M. Wu, L. Shen, C. A. Brust, S. P. Nikas, F. Song, X. Song, S. Yuan, Q. Sun, Y. Wu, S. Jiang, T. W. Grim, O. Benchama, E. L. Stahl, N. Zvonok, S. Zhao, L. M. Bohn, A. Makriyannis and Z.-J. Liu, *Cell*, 2020, **180**, 655-665.e18.

18 J. Zhang, S. Zhang, Y. Liu, M. Su, X. Ling, F. Liu, Y. Ge and M. Bai, *Photodiagnosis Photodyn. Ther.*, 2018, **24**, 185–191.

19 P. L. Prather, F. FrancisDevaraj, C. R. Dates, A. K. Greer, S. M. Bratton, B. M. Ford, L. N. Franks and A. Radominska-Pandya, *Biochem. Biophys. Res. Commun.*, 2013, **441**, 10.1016/j.bbrc.2013.10.057.
